# Distinct and overlapping control of 5-methylcytosine and 5-hydroxymethylcytosine by the TET proteins in human cancer cells

**DOI:** 10.1186/gb-2014-15-6-r81

**Published:** 2014-06-23

**Authors:** Emily L Putiri, Rochelle L Tiedemann, Joyce J Thompson, Chunsheng Liu, Thai Ho, Jeong-Hyeon Choi, Keith D Robertson

**Affiliations:** 1 Department of Molecular Pharmacology and Experimental Therapeutics and Center for Individualized Medicine, Mayo Clinic, 200 First Street SW, Rochester, MN 55905, USA; 2 Cancer Center, Georgia Regents University, 1411 Laney Walker Blvd, Augusta, GA 30912, USA; 3 Department of Hematology and Oncology, Mayo Clinic, Scottsdale, AZ 85259, USA

## Abstract

**Background:**

The TET family of dioxygenases catalyze conversion of 5-methylcytosine (5mC) to 5-hydroxymethylcytosine (5hmC), but their involvement in establishing normal 5mC patterns during mammalian development and their contributions to aberrant control of 5mC during cellular transformation remain largely unknown. We depleted TET1, TET2, and TET3 in a pluripotent embryonic carcinoma cell model and examined the impact on genome-wide 5mC, 5hmC, and transcriptional patterns.

**Results:**

TET1 depletion yields widespread reduction of 5hmC, while depletion of TET2 and TET3 reduces 5hmC at a subset of TET1 targets suggesting functional co-dependence. TET2 or TET3 depletion also causes increased 5hmC, suggesting these proteins play a major role in 5hmC removal. All TETs prevent hypermethylation throughout the genome, a finding dramatically illustrated in CpG island shores, where TET depletion results in prolific hypermethylation. Surprisingly, TETs also promote methylation, as hypomethylation was associated with 5hmC reduction. TET function is highly specific to chromatin environment: 5hmC maintenance by all TETs occurs at polycomb-marked chromatin and genes expressed at moderate levels; 5hmC removal by TET2 is associated with highly transcribed genes enriched for H3K4me3 and H3K36me3. Importantly, genes prone to hypermethylation in cancer become depleted of 5hmC with TET deficiency, suggesting that TETs normally promote 5hmC at these loci. Finally, all three TETs, but especially TET2, are required for 5hmC enrichment at enhancers, a condition necessary for expression of adjacent genes.

**Conclusions:**

These results provide novel insight into the division of labor among TET proteins and reveal important connections between TET activity, the chromatin landscape, and gene expression.

## Background

Vertebrate cellular identity arises through intricate differentiation events orchestrated by epigenetic regulation of gene expression. One key epigenetic mechanism is methylation of DNA. DNA is covalently modified by methylation of the carbon-5 position within cytosine nucleotides (5mC), an epigenetic mark that, when occurring in gene promoters, is associated with transcriptional repression. DNA methylation primarily occurs in the context of cytosine followed by guanine (CpG), and normal CpG methylation patterns have been extensively characterized in human cells [1,2]. Throughout the human genome, CpG dinucleotides tend to be methylated, except in GC-dense CpG islands (CGIs) [3-6]. For transcriptionally active genes, promoter CGIs remain unmethylated whereas intragenic domains and repetitive sequences are enriched for CpG methylation, a state that promotes genomic stability. These patterns are reversed in the cancer genome, which exhibits widespread hypomethylation and aberrant promoter CGI hypermethylation resulting in transcriptional silencing. CGI 'shores', defined as 2 kb regions that flank CGIs, also bear important epigenetic regulatory function in that they exhibit tissue-specific differential methylation that appears to regulate gene expression [7]. Furthermore, cancer genomes lose these tissue-specific patterns of CGI shore methylation, becoming either hyper- or hypomethylated in CGI shores relative to normal tissue [7].

The DNA methyltransferases (DNMTs) function in the establishment and maintenance of CpG methylation patterns. DNMT1, the 'maintenance' methyltransferase, recognizes hemi-methylated DNA for proper replication of methylation upon nascent DNA strand synthesis [8,9]. DNMT3A and DNMT3B are '*de novo*' methyltransferases, which establish new methylation patterns, especially during cellular differentiation [10-12]. Recently, the Ten-eleven translocation (TET) family of dioxygenases, TET1, TET2, and TET3, were discovered for their capacity to modulate DNA methylation patterns. The TET hydroxylases catalyze the conversion of 5-methylcytosine (5mC) to 5-hydroxymethylcytosine (5hmC) in an α-ketoglutarate- and Fe(II)-dependent manner [13,14]. In the process of demethylating DNA, TET enzymes further act on 5hmC to generate 5-formylcytosine and 5-carboxylcytosine (5caC), both of which can be removed by thymine DNA glycosylase via base excision repair [15-17]. The hydroxymethyl modification of cytosine is, however, not a rare or transient modification in the mammalian genome, with 5hmC comprising an estimated 0.6%, 0.2%, and 0.03% of total nucleotides in mouse Purkinje cells, granule neurons, and embryonic stem cells (ESCs), respectively [13,18]. This suggests that 5hmC is a stable mark, rather than a transient intermediate of cytosine demethylation. In support of this, specific genomic regions, particularly gene promoters, enhancers, and exons, are enriched for 5hmC [19-26], and binding of 5hmC by cell-specific binding partners (for example, the MBD3/NURD complex and MeCP2) shapes chromatin structure and gene expression [27-29]. Thus, if 5hmC is a stable, functional mark of the epigenome, how do the three TET proteins contribute to the patterning of 5hmC and 5mC and what is the role of this process in cancer initiation and progression?

TET1 and TET2 have been implicated in establishment and maintenance of ESC pluripotency and demethylation of the genome during somatic cell reprogramming [14,30]. Genetic disruption of *Tet1* in mouse ESCs skews differentiation toward extraembryonic lineages, but mice with a deficiency of *Tet1* and/or *Tet2* are viable, likely due to functional redundancy with *Tet3*[30-32]. *Tet3* conditional null zygotes develop to term, but neonates die postnatally at day 1 [33]. In the mouse, Tet3 is responsible for global demethylation of the male pronucleus and for zygotic epigenetic reprogramming [33-35]. Tet2 and Tet3 are also largely responsible for enrichment of 5hmC at neurodevelopmental genes during vertebrate neurogenesis, and in *Xenopus*, Tet3 is essential for expression of a set of eye developmental genes and for expression of neuronal and neural crest markers [36,37]. Taken together, the TET proteins are clearly important regulators of developmental gene expression programs and in defining normal cell identity, albeit with unique and distinct functions for each family member, which have yet to be fully characterized.

The differential functions for TET family members are also apparent in the distinct outcomes of TET mutations in human disease. Catalytic mutations in *TET2*, but not *TET1*, are commonly identified in patients with hematopoietic disorders and malignancies such as myelodysplastic syndrome, myeloproliferative neoplasms, acute myelogenous leukemia, chronic myelomonocytic leukemia, and B-cell and T-cell lymphomas [38-42]. Common among TET family members is the finding that *TET1*, *TET2*, or *TET3* mRNA and 5hmC levels are reduced across a broad spectrum of solid tumors [43-46]. Despite the revelation of widespread *TET* mutations and deregulated *TET* expression in human cancer, the effect on 5mC in these malignancies is still debated, as Ko *et al*. [47] and Figueroa *et al*. [48] observed conflicting results of 5mC changes in *TET2* mutant acute myelogenous leukemias. Likewise, our knowledge of the gene targets of TET catalytic activity is still limited. Collectively, these deficiencies hamper our understanding of the role of the TETs and 5hmC in tumor initiation and progression. In this study we systematically identify the epigenetic targets and determine the genome-wide 5mC and 5hmC patterning activities of each TET family member in human embryonic carcinoma cells by specifically depleting each TET family member using small interfering RNA (siRNA). Genes and CGIs targeted for 5hmC maintenance by TET1, TET2, and TET3 overlap extensively among the three family members, with TET1 targeting the most loci. TET1 exerts greater influence at high CpG density promoters (HCPs), while TET2 functions more prominently at low CpG density promoters (LCPs). These results reveal that TET2 and TET3 actively eliminate 5hmC, particularly in introns of highly expressed genes. The differential functions of TETs in promoting or removing 5hmC are chromatin modification specific: TET1, TET2, and TET3 enrich 5hmC at polycomb-marked H3K27me3 (histone H3 lysine 27 trimethylation) and H2AK119ub (histone H2A lysine 119 monoubiquitination) promoters and genes with moderate expression; TET2 targets H3K4me3-rich promoters and highly active genes for 5hmC removal. Depletion of the TETs resulted in large-scale hypermethylation changes, particularly within promoters and CGI shores, but TET depletion also more frequently caused hypomethylation changes of smaller magnitude in promoters and CGIs, implicating TETs in removing and promoting methylation. Importantly, enhancer enrichment of 5hmC is mediated by all three TETs and is required to promote gene expression. This study yields a comprehensive genome-wide view of TET-targeted loci in human cancer cells, revealing for the first time loci that are particularly susceptible to TET-regulated cytosine modifications and identification of distinct and overlapping functions of TET1, TET2, and TET3.

## Results

### 5hmC enrichment is associated with robust gene activation during cellular differentiation

**Figure 1:**
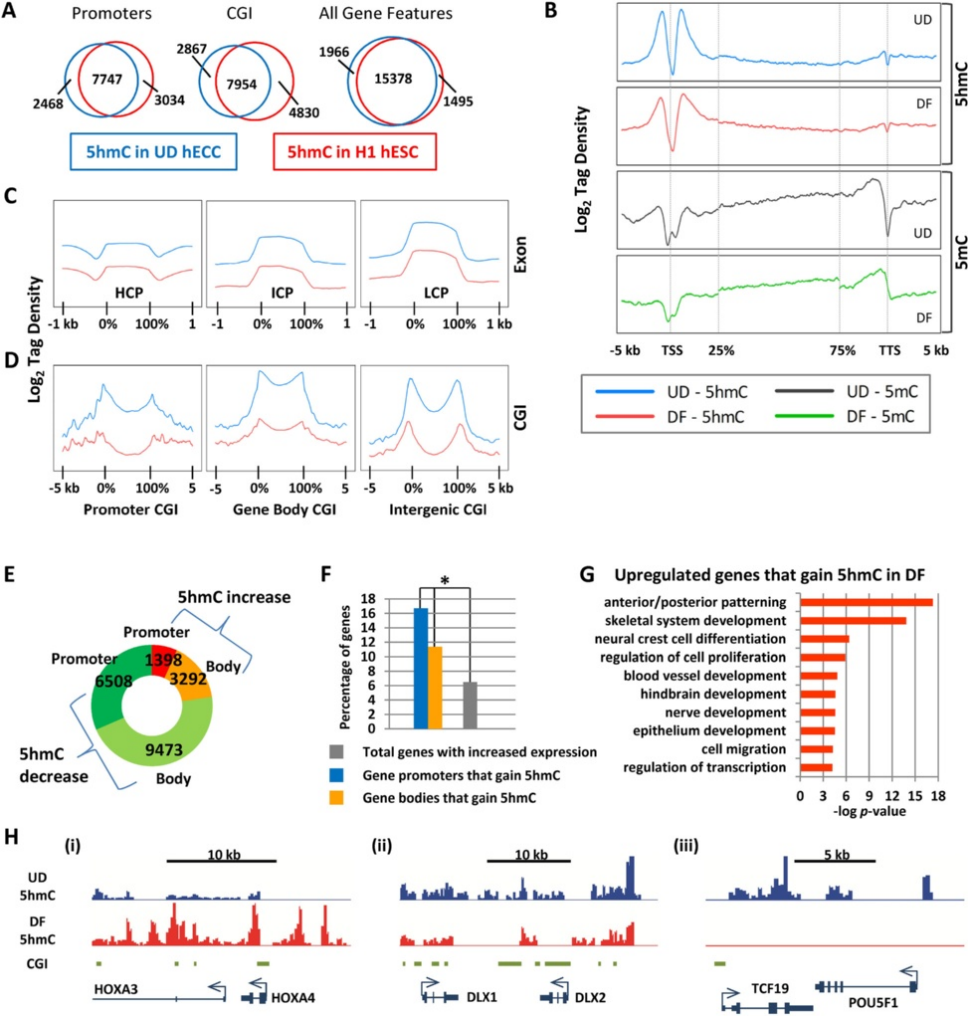
**Characterization of 5hmC patterns in undifferentiated and retinoic acid differentiated NCCIT embryonic carcinoma cells. (A)** Promoters, CGIs, and genes with peaks of 5hmC in UD NCCIT human ECCs (hECC) and H1 human ESCs (hESC) were compared. Numbers represent features common between or exclusive to UD hECCs and H1 hESCs. Overlapping sets in all three features were statistically significant (*P* < 0.0001). **(B)** Log_2_ tag density of 5hmC- and 5mC-sequencing in UD and DF cells from -5 to +5 kb across promoters, across gene bodies (represented as a percentage from 25% to 75%) and -5 to +5 kb across the TSS. Dotted lines represent TSS, +5 kb from TSS/25% of gene body, 75% of gene body/-5 kb from transcription termination site (TTS), and TTS. **(C)** 5hmC tag density across exons with high (HCP), intermediate (ICP), and low (LCP) CpG density promoters and **(D)** across CGIs in promoters, gene bodies, and intergenic regions. **(E)** Number of gene promoters and gene bodies with differential 5hmC upon RA differentiation of NCCIT cells. **(F)** Promoters and gene bodies with elevated 5hmC in DF cells were compared to genes whose expression increased upon differentiation. Blue and yellow bars represent overlapping genes with differential 5hmC and increased expression (shown as a percentage of upregulated genes); the grey bar represents the percentage of all differentiation-upregulated genes in the genome. Transcriptionally upregulated genes with gain of 5hmC are significantly overrepresented (*P < 0.0001). **(G)** Ontology analysis of upregulated genes with increased 5hmC enrichment. **(H)** Examples of the three types of 5hmC changes observed in DF cells: (i) increased 5hmC; (ii) partial loss and redistribution of 5hmC; and (iii) total or near complete loss of 5hmC.

We chose to study TET function in the human embryonic carcinoma cell (ECC) line NCCIT, which is a nonseminomatous germ cell-derived teratoma. The NCCIT expression profile resembles that of human ESCs, and NCCIT can be induced to differentiate with retinoic acid (RA) treatment into the primary embryonic germ layers and extra embryonic lineages [49]. Thus, results from this model system are potentially applicable to both ESC and cancer cell biology and to the process of differentiation. Since ECCs are less well characterized in terms of their 5hmC profile than ESCs, we compared NCCIT 5hmC and TET expression levels to other well-characterized cell/tissue types. Quantification revealed that ECCs have a 5hmC level close to that of undifferentiated human ESCs (Figure S1A in Additional file 1). NCCIT 5hmC levels are above those of an established glioma tumor cell line and well below those of normal human brain. TET expression was also examined in the same samples and showed that TET RNA levels tended to be highest in normal human brain, although there was not a perfect correlation between total 5hmC level and TET expression (Figure S1B in Additional file 1). These findings are consistent with other published studies [43]. To characterize 5hmC patterns in pluripotent NCCIT ECCs (UD = undifferentiated) and NCCIT cells differentiated with RA for 7 days (DF = differentiated), 5hmC residues were labeled with UDP-azide-glucose and biotin for affinity purification of 5hmC-containing genomic DNA fragments, followed by deep-sequencing of the 5hmC-modified DNA [50]. UD cells displayed 5hmC enrichment in gene promoters, as described for pluripotent human ESCs [51] [GEO:GSM747152]. Likewise, peaks of 5hmC enrichment occupied many of the same genomic loci in UD and H1 ESCs (*P* < 0.0001; Figure 1A) [52], reinforcing the notion that ECCs represent relevant models for ESCs and differentiation. Some promoter 5hmC enrichment (approximately 25%), however, was unique to each cell type. UD ECCs uniquely exhibited 5hmC enrichment at genes involved in neuronal differentiation and cellular morphogenesis, but lacked 5hmC enrichment at genes involved in ion transport and nucleotide metabolism (Figure S1C in Additional file 1). These differences could be attributable to differing cell of origin, degree of pluripotency, or the transformed state of the ECCs. Further validation with an independent 5hmC pull-down experiment coupled with quantitative PCR (qPCR) confirmed the NCCIT UD 5hmC-seq enrichment results at several loci (Figure S2 in Additional file 1). *HOXD10*, *HOXC5*, and *EVX2* promoters had abundant 5hmC, *HES7* and *HAND1* promoters exhibited low 5hmC levels, and the NANOG locus was devoid of 5hmC (Figure S2 in Additional file 1). In general, 5hmC accumulates in peaks flanking the TSS (transcription start site) of UD promoters (Figure 1B). Based on previously published 5mC-seq data from our laboratory in this same system [1], 5mC shows some promoter enrichment but is more abundant across the gene body toward the 3’ UTR, whereas 5hmC is relatively low throughout the gene body except near the 3’ UTR, where it shows a low level of accumulation (Figure 1B). This is in contrast to mouse ESCs in which 5hmC increases across the gene body away from the promoter [19,20]. Exons, however, display enrichment of 5hmC that is inversely proportional to promoter CpG density (Figure 1C). DF cells show similar patterns, albeit with lower levels, of 5hmC enrichment as observed in the UD state (Figure 1B). In CGIs, 5hmC density is defined by genomic location: promoter and intragenic CGIs show low levels of 5hmC, whereas gene body CGIs are 5hmC-rich (Figure 1D). Strikingly, a sharp peak of 5hmC marks the border between CGIs and CGI shores (Figure 1D). In general, 5hmC in human ECCs exhibits a distribution profile that is specific to genetic features.

Notably, most genes (approximately 80% with 5hmC changes) show a decrease of 5hmC upon induction of differentiation, but a discrete subset gain 5hmC across promoters and gene bodies based on 5hmC enrichment and deep sequencing (Figure 1E). This is consistent with the overall levels of 5hmC, which decline during NCCIT differentiation, accompanied by modest changes in TET expression. During an extended timecourse of RA-induced NCCIT differentiation, some global reduction in 5hmC was observed at day 7, the timepoint analyzed here, and 5hmC continued to decline as differentiation proceeded (Figure S1A,B in Additional file 1). Several of these changes, identified through deep sequencing, were examined at base-pair resolution by TET-assisted bisulfite conversion (TAB) coupled with Sanger sequencing [25], confirming the overall trends illustrated by the 5hmC-seq results (Figure S3A in Additional file 1). The TAB-seq results reiterate the estimate by Yu *et al*. [25] that 5hmC comprises a low amount (estimated to be 3 to 4%) of total intragenic cytosines. Expression microarrays were used to identify relationships between 5hmC and expression upon induction of the differentiation program. Genes that gain 5hmC in DF cells are significantly (*P* < 0.0001) prone to activation upon differentiation and are enriched for genes involved in patterning and differentiation of ectodermal derivatives (for example, hindbrain, nerve, and epithelium development) (Figure 1F,G). Genes with 5hmC depletion after differentiation showed a slight (but not significant: *P* = 0.1419) trend toward downregulated expression. 5hmC-depleted genes can be classified into two subsets: those with variable loss and/or redistribution of 5hmC and genes with complete loss of 5hmC (Figure 1H; Figure S4 in Additional file 1). Together, these data suggest that 5hmC is enriched at genes primed for differentiation-induced upregulation, potentially contributing to a poised chromatin state.

### Roles for TET1, TET2, and TET3 in patterning methylcytosine across intragenic regions

NCCIT ECCs serve as a model for understanding the function of TET dioxygenases in patterning the methylome because all three TET enzymes are abundantly expressed. TET3 expression is about 1.7-fold that of TET2, and TET1 is the most abundant of the three TETs, with about 30-fold the expression of TET2, a ratio similar to that observed in human ESCs (Figure S1B, right panel in Additional file 1). To investigate their functions, we depleted TET1, TET2, and TET3 by siRNA transfection in UD NCCIT cells. This method generates transient, acute depletion of each TET, allowing us to observe the most immediate, direct epigenetic effects of the functional depletion and avoiding potential compensatory changes that have been shown to occur with other methods such as transgenic small hairpin RNA or gene knockouts [53,54]. A non-targeting control (NTC) siRNA was utilized for comparison. Transcript levels for TET1, TET2, and TET3 were depleted by 60 to 70% over 72 hours (Figure S5 in Additional file 1). No phenotypic changes were observed in siTET-treated cells relative to siNTC-treated cells during the 72 hour experiment (not shown). These depletions had little effect on the transcript abundance of *DNMT1*, *DNMT3A*, or *DNMT3B* or of the other TETs. Likewise, transcription of the housekeeping genes *TUBA1C*, *DYNLL*, and *RPL30* was unaffected, showing no off-target effects and no defects in major cell processes or viability. Since 5hmC abundance was loosely connected with gene expression during differentiation in NCCIT, we asked whether TET1, TET2, or TET3 regulate the expression of pluripotency or differentiation markers. Depletion of TET transcripts did not impact lineage marker expression, except for the trophectodermal marker HAND1 (Figure S5 in Additional file 1). This result is consistent with prior studies in *Tet1*-deficient mouse ESCs that showed skewing toward trophectodermal fate [14,30,31]. To determine the impact of TET depletion, total levels of 5mC and 5hmC were assayed with 5mC- and 5hmC-specific antibodies in an ELISA-like detection assay. The genomic abundance of 5mC was not significantly affected by TET depletion (although there was a trend toward hypermethylation in TET2 and TET3 depletions; Figure S6 in Additional file 1). siTET1 cells showed approximately 60% loss of 5hmC, but siTET2 and siTET3 did not significantly impact total 5hmC (Figure S6 in Additional file 1). Thus, do each of the TETs have region-specific or site-specific impacts on 5hmC and 5mC?

**Figure 2:**
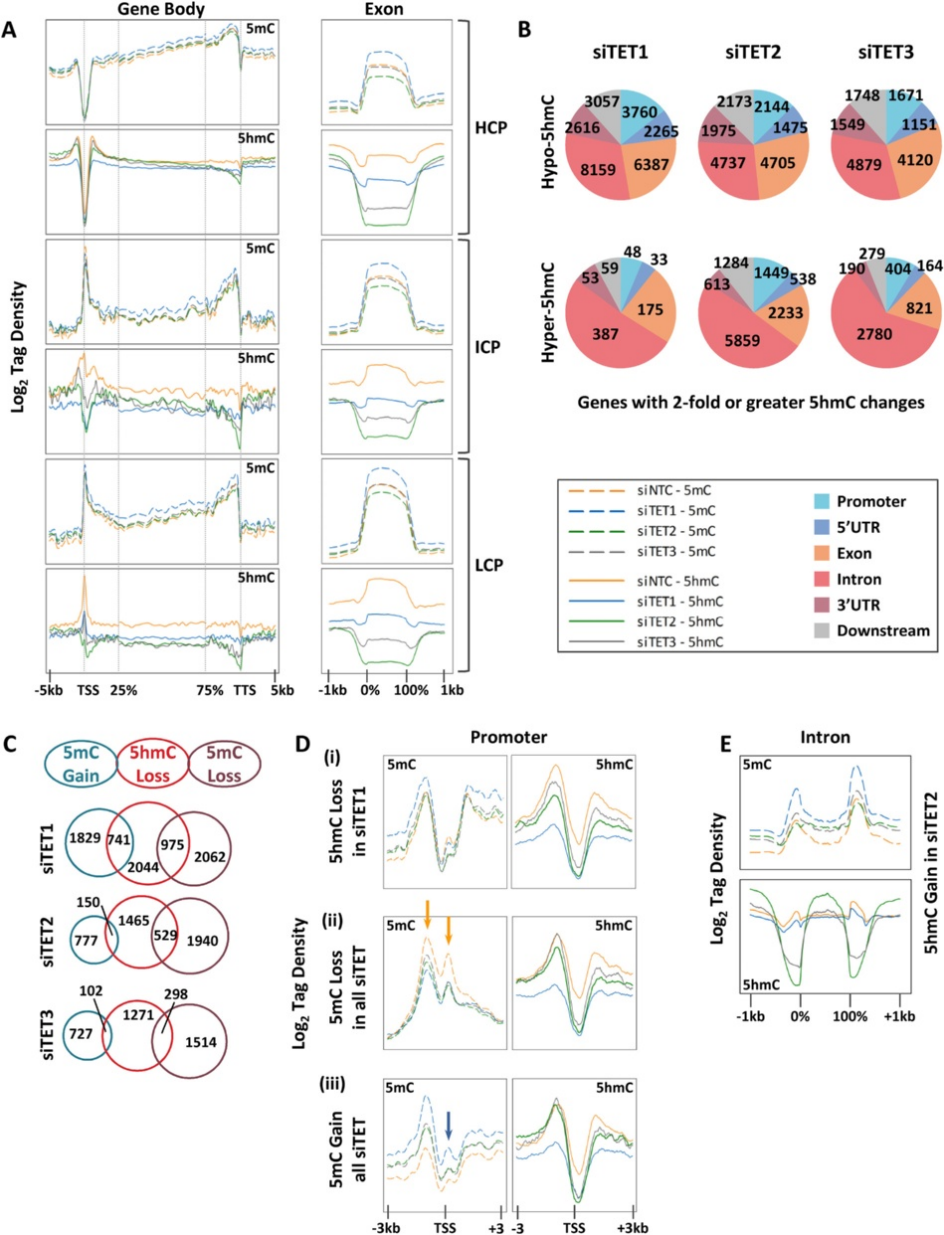
**Depletion of TET1, TET2, or TET3 causes genome-wide loss of 5hmC and both DNA hypomethylation and hypermethylation. (A)** Tag density plots of 5mC (dashed line plots) and 5hmC (solid line plots) from -5 to +5 kb across gene promoters, across gene bodies (25 to 75%), and from -5 to +5 kb across the transcription termination site (TTS) (left panels). Tag density plots were also drawn for exons across HCP, intermediate CpG density promoter (ICP), and LCP genes (right panels). **(B)** Pie charts for genes with decreased (top) and increased (bottom) 5hmC. Pie pieces represent total number of genes with two-fold or greater 5hmC change in the specified gene region. **(C)** Area proportional Venn diagrams illustrating overlap of promoters that lose 5hmC and gain or lose 5mC in each TET knockdown. *P* < 0.0001 except for overlap of TET3 hypohydroxymethylation with siTET3 hypermethylation for which *P* = 0.0009. **(D)** Tag density of 5mC (left) and 5hmC (right) for only promoters with (i) more than two-fold reduction of 5hmC in siTET1, (ii) more than two-fold reduction of 5mC in all siTET depletion conditions, and (iii) more than two-fold increase of 5mC in all siTET depletions. The region shown is -3 kb upstream and +3 kb downstream relative to TSS. Line colors are as in **(A)**. Colored arrows indicate approximately -1 kb and +250 bp positions relative to the TSS. **(E)** Tag density of 5mC (top) and 5hmC (bottom) across intronic sequences for only genes showing increased 5hmC within introns of siTET2-treated cells.

5hmC-seq and 5mC-seq were performed on siTET1-, siTET2-, and siTET3-treated cells to determine the specific roles of each TET on patterning the epigenome. Scatter plots were used to compare levels of 5hmC and 5mC peaks between siTET- and siNTC-treated cells (Figure S7A,B in Additional file 1). siTET cells had peaks with both lower and higher 5hmC levels relative to siNTC. All siTET knockdowns, but particularly siTET1, caused robust hypermethylation at sites with low to moderate basal 5mC levels (red arrow in Figure S7B in Additional file 1). This comparison also revealed some hypomethylation at sites with high basal methylation in siTET1 (green arrow in Figure S7B in Additional file 1). 5mC tag density across gene bodies shows a subtle increase in response to TET depletion, with siTET1 yielding the most hypermethylation (Figure 2A). Promoter distribution of 5hmC and 5mC is CpG density-dependent, with HCPs displaying low 5hmC and 5mC levels and LCPs being enriched for 5hmC and 5mC (Figure 2A). In 5hmC-rich promoters, exons, and 3’ UTRs, depletion of any of the TETs induced a general loss of 5hmC (Figure 2A). Reductions in 5hmC in the promoter were CpG density-dependent as noted by the 5hmC tag densities surrounding the TSS; siTET1-treated cells showed the greatest reduction of 5hmC at HCPs, whereas siTET2-treated cells displayed the greatest reduction of 5hmC at LCPs. 5hmC loss in exons was abundant in all siTET depletions, with siTET1 and siTET2 showing the least and most reduction in 5hmC, respectively (Figure 2A).

Peaks of differential 5hmC and 5mC across intragenic regions were used to assess the site-specific epigenetic effects of each TET depletion [55]. Genes with at least two-fold increase or decrease within promoters, UTRs, exons, introns, and regions 1 kb downstream of the transcription termination site (TTS) were counted (Figure 2B; Figure S7C in Additional file 1). siTET1 yielded predominantly hypo-hydroxymethylation. siTET2 and siTET3 cells developed loci with both hypo- and hyper-hydroxymethylation. Notably, in siTET2, intragenic regions tended to lose 5hmC, except introns. This effect was even more apparent when 5hmC changes were stratified by magnitude. Introns most affected by siTET2 and siTET3 (more than four-fold 5hmC changes) gained 5hmC (Figure S8A in Additional file 1). Select loci predicted to lose 5hmC based on the sequencing data were confirmed by independent 5hmC pull-down coupled with qPCR (Figure S9 in Additional file 1). Analysis of differential 5mC peaks confirmed our earlier observation (Figure S7B in Additional file 1) that both hypermethylation and hypomethylation result from siTET depletion (Figure S8B in Additional file 1). Intriguingly, the most robust (more than four-fold) 5mC changes were hypermethylation events. Numerous smaller 5mC changes of less than four-fold were most frequently hypomethylation events (Figure S8B in Additional file 1; Additional file 2). Thus, depletion of TET1, TET2, or TET3 caused hypomethylation events of small magnitude and hypermethylation events of larger magnitude. Both hypomethylation and hypermethylation changes were significantly enriched at loci that lost 5hmC in siTET1, siTET2, and siTET3 cells (Figure 2C), linking the two opposing outcomes. These results, taken together with the results in Figure S6 in Additional file 1 showing no net gain or loss of total 5mC, suggest that 5hmC depletion in siTET knockdown cells leads not to global hypermethylation but instead to a redistribution of global 5mC.

Promoters with decreased 5hmC overlapped extensively among the TET knockdowns (58 to 90% overlap), showing overlapping function of TET1, TET2, and TET3 at these loci (Figure S10A, left in Additional file 1); TET1 showed the largest number of unique targets with hypo-hydroxymethylation. 5hmC-depleted promoters in siTET1, siTET2, or siTET3 cells represented genes with roles in embryonic development, cell adhesion, motility, and proliferation (Figure S10B in Additional file 1) and corresponded highly with those promoters that lose 5hmC upon differentiation of NCCIT cells (approximately 60% of siTET targets overlap with DF-induced 5hmC changes; *P* < 0.0001; Figure S10A, right in Additional file 1). Thus, TET1, TET2, and TET3 co-regulate cytosine modifications at many of the same target sites, and these co-regulated targets control embryonic development and basic cellular physiology. In addition, our results clearly show that neither DNA hypermethylation nor hypo-hydroxymethylation is the sole outcome of TET depletion, suggesting that the role of the TETs in regulating DNA methylation is more complex than previously thought.We next asked how loss of 5hmC impacts 5mC distribution around the TSS by plotting the tag density for only genes with 5hmC loss in siTET1-treated cells. These loci showed a large trough of 5mC across the TSS, but TET depletion did not impact the overall 5mC distribution at these promoters that lose 5hmC (Figure 2D(i)). Similarly, we plotted the 5mC distribution for subsets of genes that lose (Figure 2D(ii)) and gain (Figure 2D(iii)) 5mC in all siTET cells (Figure 2D). Hypomethylated promoters display peaks of 5mC at -1 kb upstream of the TSS and immediately downstream of the TSS (Figure 2D(ii), orange arrows). Hypomethylation at these promoters is subtle and occurs in the immediate vicinity of the TSS (Figure 2D(ii)), whereas promoter hypermethylation is much more dramatic and occurs across a >6 kb region flanking the TSS (Figure 2D(iii)). Hypermethylated promoters also have a peak of 5mC at -1 kb (albeit, not as pronounced as that in hypomethylated promoters) but display a distinct depression of 5mC between -250 bp to +750 bp surrounding the TSS (Figure 2D(iii), blue arrow). In the siTET-treated cells, the peak of 5mC at -1 kb increases, and the depression across the TSS regains a peak of 5mC. Thus, the methylation landscape in promoters is dramatically different for those loci that become hypomethylated versus those that become hypermethylated upon TET depletion. Since TET2 depletion resulted in 5hmC enrichment particularly in introns, we plotted the 5hmC and 5mC tag density for introns with hyper-5hmC. These genes showed substantial redistribution of 5hmC patterns (Figure 2E). siNTC and siTET1 introns had low invariable 5hmC across introns, but siTET2 and to a lesser extent siTET3 showed a striking peak of 5hmC across introns typically associated with 5hmC depletion in flanking exons.

### TET proteins control cytosine modifications at enhancers and prevent hypermethylation of promoter CGI shores

**Figure 3:**
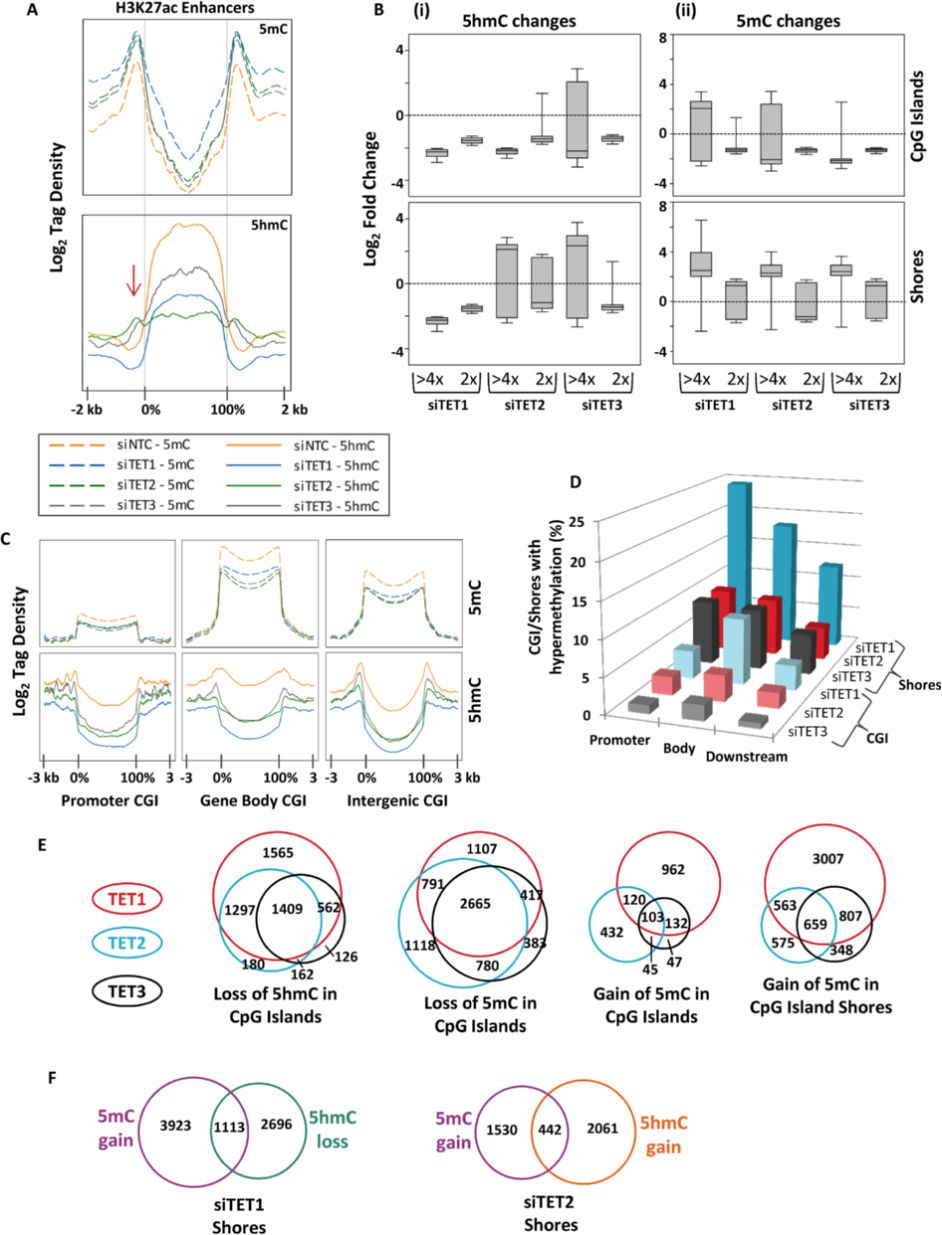
**Impact of TET depletion at key regulatory elements. (A)** Tag density plots of 5mC (top) and 5hmC (bottom) in enhancer elements defined by previously published H9 ESC H3K27ac ChIP-seq profiles [GEO: GSM605307]. Red arrow in the bottom panel denotes a trough of 5hmC at enhancer boundaries that is lost in siTET2/3-treated cells. **(B)** Box plots of log_2_ fold-change based on differential SICER analysis of (i) 5hmC and (ii) 5mC peaks. Fold-change is shown for CGIs and CGI shores and is stratified by changes of greater than four-fold (>4×) and changes between two-fold and four-fold (2×). **(C)** Tag density plots of 5hmC and 5mC for CGIs in promoters, gene bodies, and intergenic regions. **(D)** Bar graph illustrating the proportion of CGIs and CGI shores in three gene regions that sustain hypermethylation in each TET knockdown. **(E)** Area proportional Venn diagrams of CGIs with loss of 5hmC, loss of 5mC, and gain of 5mC under siTET1, siTET2, and siTET3 depletion conditions. **(F)** Area proportional Venn diagrams representing CGI shore hypermethylation coinciding with 5hmC gain in siTET2 and 5hmC loss in siTET1 depletion conditions (*P* < 0.0001 for both).

Previous genome-wide profiling of 5hmC showed that this mark was enriched at enhancers, although the role of each TET in mediating this was not examined [24-26]. Using profiles for acetylation of H3K27 (H3K27ac) in H9 ESCs as enhancer annotations [56] [GEO:GSM605307], we examined 5hmC abundance in NCCIT cells. In siNTC-NCCIT cells 5mC and 5hmC show an inverse enrichment: 5mC is low inside enhancers but enriched at their boundaries (Figure 3A, top); 5hmC is strongly enriched within enhancers but falls at the boundaries forming a sort of gutter at the enhancer edge (Figure 3A, bottom). TET2 depletion had the greatest impact on average 5hmC at enhancers (Figure 3A, bottom), but TET1 targets a greater number of enhancer elements for 5hmC enrichment (Figure S11 in Additional file 1). 5hmC in enhancers is mostly depleted in siTET2 and is partially depleted in siTET1 and siTET3 conditions, indicating involvement of these TETs, but especially TET2, in establishing and/or maintaining 5hmC at enhancers. The gutter of 5hmC at enhancer boundaries in siTET2- and siTET3-treated cells recedes, resulting in 5hmC accumulation, suggesting that TET2 and TET3 may help to define enhancer borders (Figure 3A, red arrow).

CGIs experienced 5hmC depletion in siTET1-, siTET2-, and siTET3-treated cells, but a large proportion of shores had elevated 5hmC levels in siTET2- and siTET3-treated cells (Figure 3B(i); Figure S12A(i) in Additional file 1). The median 5mC changes in CGIs for siTET1 and siTET2 were hypomethylation (Figure 3B(ii); Figure S12A(ii) in Additional file 1). This is in stark contrast to CGI shores, which were robustly hypermethylated in siTET1, siTET2, and siTET3 cells (Figure 3B(ii)). CGI methylation patterns occurred irrespective of intragenic versus intergenic location (Figure 3C); however CGI shore hypermethylation was most abundant in shores associated with promoters, as 26%, 10%, and 11% of gene promoters with CGIs had hypermethylated shores upon TET1, TET2, and TET3 depletion, respectively (Figure 3D). CGIs that lose 5mC or 5hmC significantly overlap among the TET knockdowns (Figure 3E; Figure S12B,C in Additional file 1), but analysis of CGI and CGI shore hypermethylation events reveals unique targets between TET1 and TET2 (Figure 3E). A significant proportion of hypermethylated CGI shores in siTET1 cells had decreased 5hmC (Figure 3F). On the other hand, CGI shore hypermethylation in siTET2 cells was associated with increased 5hmC (Figure 3F). TET1 targets promoter CGI shore hypermethylation at genes involved in basic cellular processes such as intracellular transport, transcription, and cell death (Figure S12D in Additional file 1). TET2 targets promoter CGI shore hypermethylation at genes involved in cytoskeletal organization, cell signal transduction pathways, and morphogenesis (Figure S12D in Additional file 1). Thus, again, TET1 and TET2 demonstrate a functional divergence in their impact on the epigenome. In summary, TET1, TET2, and TET3 preferentially remove methylation at CGI shores, particularly those within promoters, suggesting that TET activity is heavily influenced by CpG density, and TET1 and TET2 target separate sets of CGI shores where they function exclusively of one another in 5mC removal.

### Gene body hypomethylation in siTET depletion conditions is associated with gene repression

**Figure 4:**
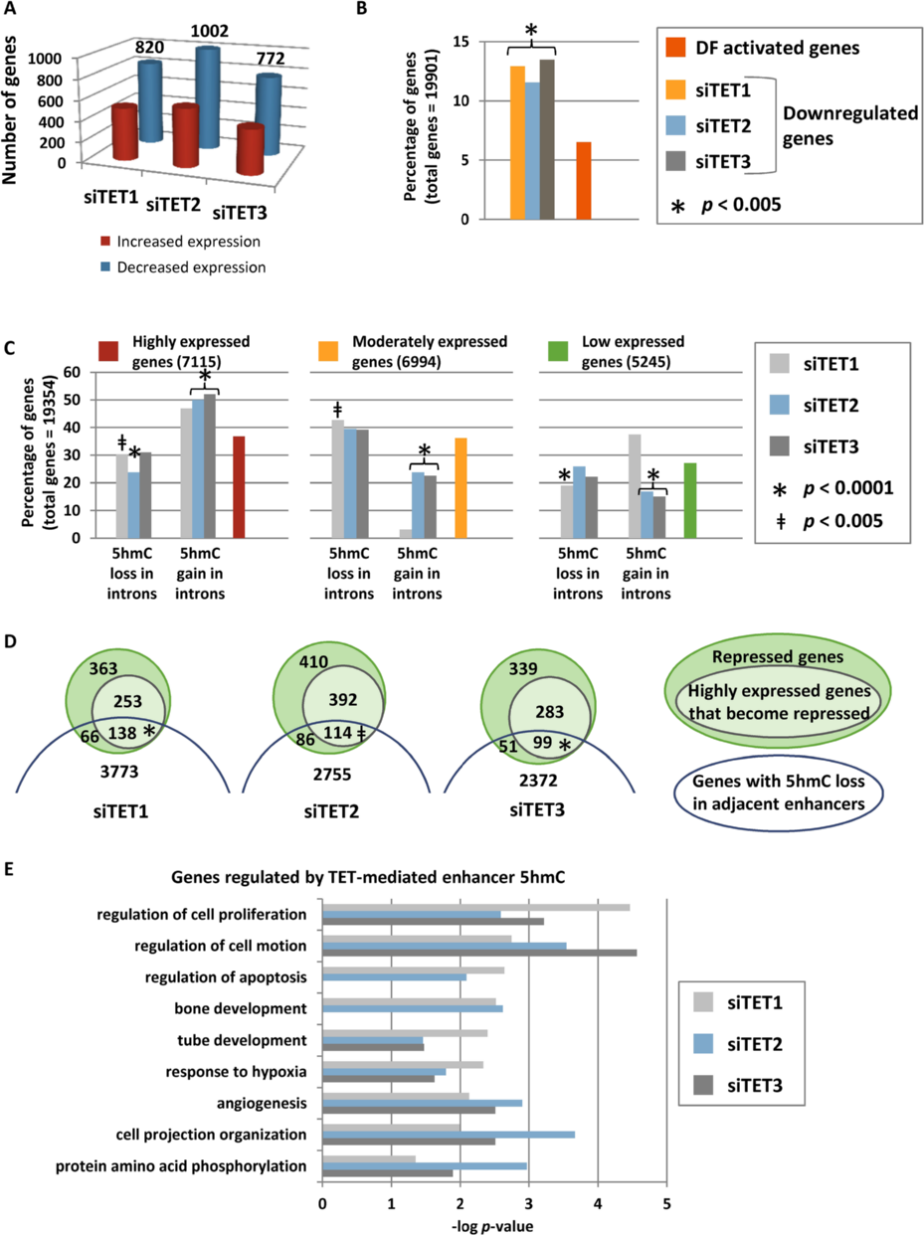
**Relationships between gene expression and DNA epigenetic marks. (A)** Quantity of upregulated and downregulated genes in siTET1, siTET2, and siTET3 conditions. **(B)** NCCIT cell gene expression changes that occur with siTET depletions were compared to gene expression changes that occur during RA-induced differentiation from UD to DF. Downregulated genes in siTET1, siTET2, and siTET3 depleted cells were significantly enriched for genes upregulated during differentiation. Shown are the percentages of genes downregulated in siTET conditions that overlap with genes that become upregulated in DF cells and the percentage of total upregulated genes in DF cells. **(C)** Hypo- or hyper-5hmC introns of siTET depletions were compared to basal gene expression levels in UD NCCIT cells. Shown is the percentage of genes with 5hmC changes in introns in siTET-treated cells that occur in highly, moderately, or lowly expressed genes. These percentages of overlapping genes are compared to the total percentage of highly (red bar), moderately (gold bar), or lowly (green bar) expressed genes. **(D)** Area proportional Venn diagrams showing the number of repressed genes that are in proximity to H3K27ac-marked enhancers that lose 5hmC upon TET depletion (*P < 0.0001; ^‡^*P* = 0.0060). **(E)** Ontology analysis of repressed genes that lose 5hmC in nearby enhancers in siTET1-, siTET2-, and siTET3-treated cells.

To understand how TET depletion impacts gene expression, microarray analysis was performed for siTET1, siTET2, and siTET3 depletion and compared to siNTC under undifferentiated conditions. All three siTET depletions yielded abundant gene activation and repression events, but gene repression dominated (Figure 4A). Repressed genes were enriched for a subset of genes that become transcriptionally activated upon differentiation of NCCIT cells (Figure 4B). We next examined the relationship between gene expression and epigenetic changes in NCCIT cells. In previous reports, gene expression and methylation changes in TET1-depleted mouse ESCs did not strongly correlate, likely due to TET function independent of TET1’s catalytic domain [20]. In our study, a gene’s basal expression level largely determined the epigenetic outcome of siTET depletion. Highly transcriptionally active genes were significantly prone to increased 5hmC in introns and exons after TET2 or TET3 depletion (Figure 4C; Figure S13 in Additional file 1). Moderately expressed genes underwent 5hmC depletion under siTET1 conditions, but transcriptionally silenced/low expressed genes were excluded from 5hmC changes (Figure 4C; Figure S13 in Additional file 1). 5hmC changes in gene promoters, bodies, or associated CGIs were not associated with gene expression changes (data not shown); however, when we assessed the impact of 5hmC loss in an enhancer on its closest neighboring gene (within a 20 kb limit), there was a significant association with gene repression, particularly of genes with high basal levels of expression (Figure 4D). Approximately 20% of gene repression events under TET depletion conditions were accounted for by loss of 5hmC in an adjacent enhancer (Figure 4D). On the other hand, 5hmC increases in enhancers or gene bodies did not correlate with expression changes (data not shown). TET1, TET2, and TET3 co-mediated 5hmC enrichment at enhancers regulating expression of genes involved in cell proliferation, cell motility, and angiogenesis; TET1- and TET2-mediated 5hmC enrichment impacted genes required for apoptosis (Figure 4E). In summary, intragenic TET-mediated 5hmC enrichment is impacted by basal expression level, and most importantly, TET1, TET2, and TET3 drive gene expression by promoting hydroxymethylcytosine within enhancer elements.

### TET functions in cytosine modification at active and repressed chromatin

**Figure 5:**
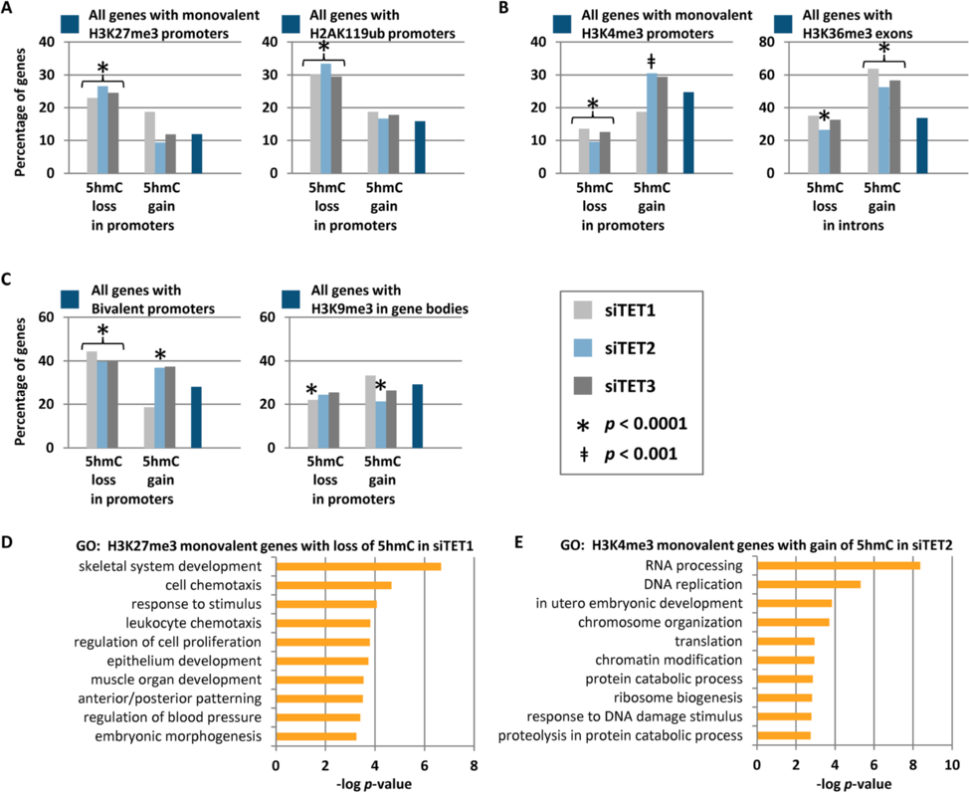
**Relationships between hydroxymethylation changes under TET depletion conditions and histone mark occupancy. (A-C)** Genes with changes in 5hmC after TET depletion were compared to subsets of genes with histone modifications (as marked) mapped previously in UD NCCIT cells [1]. Shown are the percentage of genes with 5hmC changes that overlap with the given histone modification, and the percentage of all promoters (or other features) with the given histone modification in the genome (total number of genes = 23,218; total genes with 5hmC changes per region are as listed in Figure 2B). 5hmC changes that have an overrepresentation or underrepresentation of the given histone mark are designated: *P < 0.0001 or ^‡^*P* < 0.001. **(D)** Ontology analyses of subsets of H3K27me3-marked genes with loss of 5hmC after siTET1 treatment and of **(E)** H3K4me3-marked genes with gain of 5hmC after siTET2 treatment.

We examined how TET1, TET2, and TET3 interface with epigenetic marks characteristic of different chromatin domains (that is, active, repressed, or transcriptionally poised). Our group has previously assessed histone mark occupancy in UD and DF NCCIT cells [1]. Genes with H3K4me3, H3K27me3, or H2AK119ub-marked promoters were compared with those genes that sustain hypo- and hyper-5hmC events in siTET depletion conditions. H3K27me3- and/or H2AK119ub-marked promoters tend to lose 5hmC in the absence of TETs, suggesting that TET1, TET2, and TET3 promote 5hmC accumulation at these loci (Figure 5A). H3K4me3-monovalent promoters, which typically lack 5hmC but are occupied by TET1 [20,21], show a propensity for 5hmC accruement in siTET2-treated cells but are protected from loss of 5hmC in all TET-depleted cells (Figure 5B). Along these lines, a significant proportion of genes enriched for H3K36me3 in exons, a mark of transcriptional activity, undergo hyper-5hmC in introns of siTET1-, siTET2-, and siTET3-treated cells (Figure 5B). Together these results indicate that TET proteins, especially TET2, engage in removing 5hmC from genes within chromatin marked for transcriptional activity. Bivalent promoters (containing both H3K4me3 and H3K27me3), which are 5hmC-rich, lose 5hmC when any of the TETs are depleted, but a subset of these loci are also prone to gain 5hmC under TET2- or TET3-depletion conditions (Figure 5C). Lastly, genes with H3K9me3 marks, which are associated with heterochromatin and transcriptional silencing and are devoid of 5hmC in normal cells, were significantly excluded from 5hmC changes in TET1- and TET2-depleted cells (Figure 5C). H3K27me3-marked genes that lose 5hmC after siTET1 treatment function in developmental processes such as patterning and morphogenesis (Figure 5D). H3K4me3-marked genes with increased 5hmC after siTET2 treatment drive basic cellular processes such as DNA replication, RNA processing, and translation (Figure 5E).

Hypomethylation and hypermethylation outcomes were also closely connected with chromatin domains. H3K27me3-marked promoters and H2AK119ub-marked promoters were susceptible to hypomethylation upon TET depletion (Figure S14A in Additional file 1). Intriguingly, promoter hypermethylation also tended to occur at H2AK119ub-marked promoters (but not H3K27me3, H3K4me3, or bivalent promoters), suggesting that TETs actively demethylate 5mC at H2AK119ub-marked promoters. H3K4me3-marked promoters were protected from hypo- and hypermethylation under TET-depletion conditions, and genes marked with exon H3K36me3 were prone to intragenic hypermethylation (Figure S14B in Additional file 1). Generally, bivalent promoters and H3K9me3-marked genes were not targeted for hypo- or hypermethylation by TET proteins (in fact, H3K9me3-marked genes were significantly protected from methylation changes; Figure S14C in Additional file 1). In summary, H3K27me3- and H2K119Aub-marked genes tend to lose both 5hmC and 5mC when TETs are depleted. Active H3K4me3- and H3K36me3-marked genes become enriched for 5hmC in promoters and exons and become hypermethylated in gene bodies upon TET depletion. Thus, polycomb-repressed genes and highly active, H3K4me3/H3K36me3-marked genes exhibit opposing epigenetic fates under TET depletion conditions, and both epigenetic fates impact pathways associated with cancer phenotypes.

### Loss of 5hmC in TET-depleted cells coincides with genes susceptible to aberrant hypermethylation in cancer

**Figure 6:**
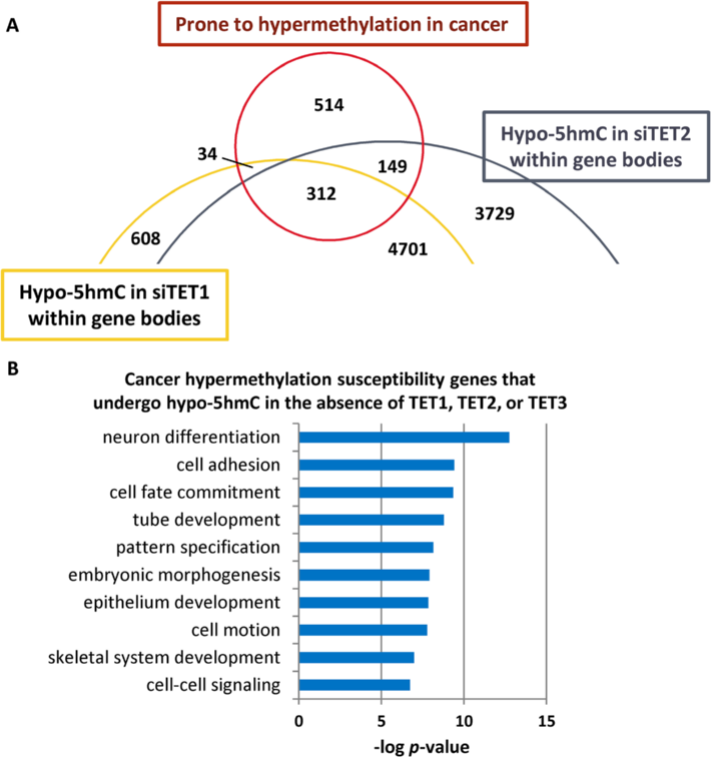
**Links between aberrant cancer methylation and TET function. (A)** Genes susceptible to hypermethylation in cancer (n = 1,009) [58] showed significant overlap with those that lose 5hmC within gene bodies (that is, exons and introns) in each of the siTET-treated conditions: 45.7% of hypermethylated genes overlap 5hmC loss in siTET1-treated cells (*P* = 0.0255); 34.3% of hypermethylated genes overlap 5hmC loss in siTET2-treated cells (*P* = 0.0002); 31.0% of hypermethylated genes overlap 5hmC loss in siTET3-treated cells (*P* = 0.0082; siTET3 not shown). **(B)** Ontology analysis of genes susceptible to promoter hypermethylation that also lose 5hmC in siTET-treated cells.

Given that aberrant promoter hypermethylation is a feature of cancer cells and a popular paradigm of TET function is to prevent this event [57], we asked whether there is any link between cancer hypermethylated loci and genes that sustain 5hmC changes in our TET knockdowns. Using a recently published list of genes that are frequently hypermethylated across a wide spectrum of human cancers [58], we investigated their relationship with genes that sustain cytosine modification changes in siTET-treated cells. Interestingly, genes susceptible to promoter hypermethylation in cancer were overrepresented among genes that lost 5hmC in siTET1-, siTET2-, and siTET3-treated cells (Figure 6A; data not shown). Likewise, siTET2 induced gain of 5hmC inversely associated with cancer hypermethylation (*P* = 0.0220; data not shown). Hypermethylation-susceptible genes with loss of 5hmC in siTET-treated cells were heavily enriched for processes involved in embryonic development, including cell fate specification, morphogenesis, and patterning (Figure 6B). In summary, TET1, TET2, and TET3 are required for maintenance of 5hmC at genes susceptible to hypermethylation in cancer. We propose that intragenic 5hmC enrichment (which was observed at genes robustly activated upon differentiation (Figure 1F)) is lost at highly transcriptionally active genes (Figure 4C) and polycomb-regulated loci upon TET knockdown (Figure 5A), permitting 5mC redistribution. 5hmC is also depleted at genes silenced by aberrant hypermethylation in cancer upon TET knockdown (Figure 6A). Enrichment of 5hmC at promoters and/or gene bodies may create a metastable transcriptionally permissive state (neither highly active nor transcriptionally silent, but capable of a robust response to stimuli); the metastable state is more easily perturbed and one consequence of such perturbation is loss of 5hmC enrichment, which may represent a crucial precursor to gene silencing by aberrant DNA methylation.

## Discussion

This study represents the first comprehensive genome-wide analysis of the role of TET1, TET2, and TET3 in patterning the distribution of 5mC and 5hmC in human cancer cells. Depletion of only one TET family member yielded robust reduction of 5hmC across intragenic regions, enhancers, and CGIs, and many of the same loci were affected by siTET1, siTET2, or siTET3 depletion conditions, suggesting a synergistic role for the TETs in establishment of 5hmC patterns in NCCIT cells. Loci uniquely affected by depletion of each TET family member were also identified. Importantly, our results reveal that TET2 and TET3, but not TET1, actively eliminate 5hmC throughout the genome, particularly at introns, as evidenced by hyper-hydroxymethylation in TET2- and TET3-depleted cells. Thus, our results suggest that all TETs, especially TET1, target loci for hydroxylation of 5mC to 5hmC, but only TET2 and TET3 are responsible for subsequent removal of 5hmC in the cytosine demethylation cascade. TET function in demethylation was particularly prominent at CGI shores, which became disproportionately hypermethylated relative to CGIs and surrounding regions. In addition to the role of the TETs in DNA demethylation, the results of this study unexpectedly implicate TETs in promoting DNA methylation, and show that TET activity is also closely connected with specific chromatin domains. The finding that TETs have a role in promoting 5hmC at loci targeted for aberrant methylation in cancer is consistent with an overall observation that TET establishment of intragenic 5hmC enrichment is associated with a state of transcriptional permissiveness. Likewise, TET-mediated 5hmC at enhancers is crucial for expression of neighboring highly active genes.

One key finding from our results was that genes and CGIs targeted for 5hmC maintenance by TET1, TET2, and TET3 overlap extensively among the three family members, but the impact of each TET on target gene DNA methylation was CpG density-dependent. Promoter 5hmC levels are inversely correlated with CpG density, as low CpG density promoters show the most abundant 5hmC in UD NCCIT cells, similar to murine ESCs [23]. Depletion of TET1 had the greatest impact on 5hmC loss at HCPs, also consistent with results in murine ESCs where Tet1 was genetically inactivated [59]. TET2 depletion in our system, however, had the greatest impact on 5hmC loss at LCPs, suggesting that TET1 and TET2 function more prominently at HCPs and LCPs, respectively. Such functional divergence between TET1 and TET2 likely relates to their protein structure. TET1 contains a CXXC zinc finger domain and possesses high affinity for non-methylated CpG-dense regions [20,60,61], whereas TET2 lacks this motif, perhaps allowing it to function more readily at or be specifically targeted to regions of low CpG density.

Reduction of TET2 or TET3 levels caused 5hmC accumulation in many regions of the genome. The simplest explanation for this observation is that TET2 and TET3 are primarily responsible for the formation of downstream cytosine intermediates (that is, 5-formylcytosine and 5-carboxylcytosine) within the demethylation cascade and disruption of either TET2 or TET3 yields accumulation of 5hmC, although this hypothesis has yet to be tested directly. An alternative explanation is that TET2 and TET3 limit each other’s 5mC to 5hmC hydroxylation activity. Within gene bodies, 5hmC accumulation was particularly evident in introns. In UD NCCIT cells, exons are enriched for 5hmC over introns, and these results suggest a role for TET2 and TET3 in the removal of 5hmC from introns. The potential impact of this function on maintaining the rate of transcription, preventing spurious transcription initiation, or preserving splicing fidelity is intriguing but unknown.

**Figure 7:**
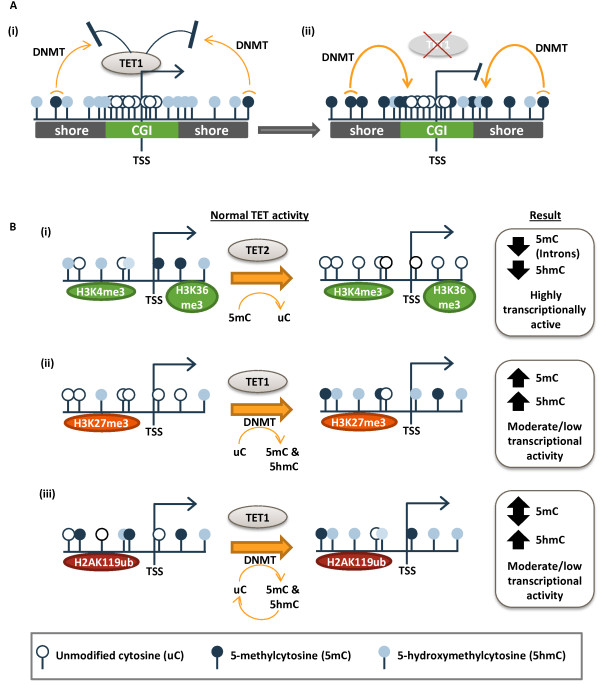
**Models for the multi-dimensional functions of TETs in mediating DNA methylation and hydroxymethylation. (A)** TET family members enrich 5hmC at CGI shores to provide a protective boundary against aberrant hypermethylation (i). In the absence of TETs, 5hmC cannot be established, permitting the aberrant expansion of 5mC into the CGI (ii). **(B)** In the normal pluripotent state, TETs eliminate 5hmC from promoters and remove both 5mC and 5hmC from gene bodies of a subset of highly transcriptionally active H3K4me3- and H3K36me3-marked genes (i). TETs mediate hydroxymethylation and promote a low level of methylation at H3K27me3-marked promoters (ii). At H2AK119ub-marked promoters TET proteins enrich 5hmC and promote turnover of cytosine modifications by mediating demethylation of 5mC (iii). In the event of TET mutation or other TET functional disruption (like decreased expression), these distinct epigenetic patterns are lost, making loci vulnerable to changes in transcriptional activity, perhaps by leaving unmodified cytosines available for aberrant DNA methylation by DNMTs or by permitting the binding of chromatin remodelers or repressors such as PRC1 or PRC2.

Some promoters targeted by the TETs for establishment of 5hmC exhibited large-scale DNA hypermethylation upon siRNA depletion. This is consistent with a study where Tet1 was depleted in murine ESCs, which resulted in increased 5mC in a subset of promoters [59]. This current analysis also includes siTET2 and siTET3, and reveals that all three TET family members are essential for promoter demethylation in the cancer genome. Unexpectedly, we observed that promoter and CGI hypomethylation events (of relatively small magnitude) far outnumbered promoter hypermethylation events (which tended to be of larger magnitude). As we discuss further below, DNA hypomethylation resulting from TET depletion may be an indirect consequence of reduced 5hmC or the result of TET functions that are separate from their known catalytic activity on cytosine substrates. Interestingly, CGI shores showed dramatic hypermethylation upon TET knockdown, especially for siTET1, and this was particularly evident for CGIs in promoters (Figure 7A). CGIs and CGI shores within promoters have a very low basal level of DNA methylation but display enrichment of hydroxymethylation at the island-shore border (Figure 7A(i)). We propose that hydroxymethylation at CGI shores establishes a protective boundary for maintenance of methylation-free CGI promoters. Possible mechanisms include creation of a chromatin configuration that blocks DNMT access, establishment of a zone where any 5mC is rapidly converted to 5hmC, or through an impact on the binding of boundary factors like CTCF [62]. When these boundaries become compromised in the absence of TET (TET downregulation or mutation), either through loss of 5hmC and/or the chromatin-bound TET proteins themselves, aberrant CGI shore hypermethylation accumulates and expands throughout the CGI, converting the transcriptionally permissive state to one that can no longer respond to stimuli (Figure 7A(ii)). Elucidation of CGIs at greatest risk for this event might be revealed by depleting or inhibiting the TETs for a longer time period.

This study also reveals a dynamic interplay between TET activity and different chromatin marks. Previous work elucidated an association between TET1/5hmC occupancy and polycomb-/trithorax-mediated histone marks in ESCs [19-21], but our study is the first to provide a functional assessment of TET1, TET2, and TET3 activities within different chromatin domains in cancer cells for which extensive chromatin mark mapping is also available. These results indicate two opposing functions for TETs at H3K4me3-marked promoters and polycomb-marked (H3K27me3 and H2AK119ub) promoters: removal of 5hmC and enrichment of 5hmC, respectively (Figure 7B). H3K4me3-marked promoters are enriched for TET1 binding but are devoid of 5mC or 5hmC [19-21]. H3K4me3-marks are also characteristic of highly expressed genes and are typically associated with gene body H3K36me3 enrichment. The results herein reveal that TET2, in particular, is responsible for eliminating 5hmC at promoters of these highly expressed, H3K4me3- and H3K36me3-marked loci (Figure 7B(i)), providing an explanation for the paradoxical observation that H3K4me3-marked promoters are TET1-rich but 5hmC-deficient. TET1 was proposed to protect active H3K4me3 monovalent promoters from aberrant hypermethylation [19-21]. In our study, however, even with depletion of TET1, TET2, or TET3, H3K4me3-marked sites remained protected from DNA hypermethylation, suggesting that each of the TETs is dispensable for maintaining hypomethylation at H3K4me3 loci or that there is functional redundancy among the TETs in this protective role. 5hmC-rich H3K4me3- and H3K27me3-marked bivalent promoters showed significant 5hmC loss and gain in response to depletion of each of the TETs. Interestingly, bivalent promoters did not significantly accumulate or lose 5mC in response to TET depletion. One possible explanation for this is that TETs stabilize 5hmC (and possibly the other demethylation intermediates) but do not actively convert 5mC to 5hmC at bivalent promoters. This is further substantiated by unpublished data from our laboratory that DNMTs have no or low activity at bivalent promoters in the pluripotent state, suggesting that bivalent promoters have neither a propensity for 5mC accumulation nor a basis for active demethylation.

Polycomb repressive complex 2 (PRC2) recruits TET1 to bivalent H3K4me3- and H3K27me3-marked promoters, which are 5hmC-rich [63]. H2AK119ub- and H3K27me3-marked promoters showed a disproportionate loss of 5hmC and 5mC under TET knockdown conditions, suggesting that TET1, TET2, and TET3 establish these cytosine modifications at polycomb repressed loci, perhaps as a means of mediating repression of PcG target genes by supporting 5mC accumulation (Figure 7B(ii,iii)). The facilitation of DNA methylation by TETs may occur indirectly and independently of their catalytic activity by facilitating a repressive chromatin state. Along these lines, TET1 recruits SIN3A, a known DNMT3B-interacting partner, to a subset of TET1 target genes (including H3K27me3-positive loci), thereby mediating transcriptional repression and possibly potentiating DNMT3B-dependent methylation [20,64]. This model is supported by our laboratory’s observation that TET-depletion-induced hypomethylation occurred at a subset of promoters occupied by DNMT1 or DNMT3B (data not shown), again, pointing to a role for TETs in facilitating cytosine methylation. Since many promoters with loss of 5mC also exhibited loss of 5hmC in siTET cells, TETs might promote cytosine methylation through establishment of 5hmC. Intriguingly, UHRF1 binds 5hmC with high affinity, and UHRF1 is required for maintenance of DNA methylation by DNMT1 during DNA replication [29,65]; thus, UHRF1 may provide a mechanistic link in this relationship in that 5hmC accumulation acts not as a precursor for active DNA demethylation, but rather is a signal for *de novo* DNA methylation. It will be critical in future experiments to determine the turnover rate of methyl groups and their derivatives in different chromatin domains of the genome, and correlate this with the presence or absence of DNMTs and TETs. Regions may exist where methyl group turnover promotes DNA demethylation, perhaps due to a lack of DNMTs, high TET-directed oxidation activity, and/or a particular combination of histone modifications. Alternatively, loci may exist where methyl group turnover combined with high DNMT activity, repressive chromatin signatures, and as yet uncharacterized TET activities (perhaps independent of their known catalytic functions), is essential for new or more extensive DNA methylation marks. If methyl group turnover is common even in constitutively methylated regions of the genome, then deregulation of any of the steps in this pathway (DNMTs, TETs, interacting factors, and substrate availability) could contribute to the hypo- and hypermethylation events that typify cancer cells.

Our expression analyses revealed both transcriptional activation and repression events resulting from depletion of each TET. Other examples of Tet1 depletion in murine ESCs have demonstrated upregulation and downregulation of target genes, indicating both transcriptional activation and repression roles for Tet1 [20,59]. In all siTET depletions, transcriptional repression was significantly linked with loss of 5hmC at adjacent H3K27ac-marked enhancers, providing direct evidence that maintenance of 5hmC in enhancers is required to drive gene expression, particularly for highly expressed genes. Expression changes induced by TET deficiency did not show a clear relationship with promoter methylation changes (that is, gene repression did not significantly correspond with promoter hypermethylation). This is not completely unexpected, given previous studies showing that expression changes in siTet1-treated DNMT triple knockout cells, which have no 5mC or 5hmC, were similar to expression effects in siTet1-treated DNMT wild-type cells, suggesting that, for some genes, the impact of Tet1 on expression is independent of its catalytic activity [20] and may be due to the TET1 protein itself or other uncharacterized ‘activities’ of TET1. Thus, we suspect that some of the expression changes observed in our siTET1-, siTET2-, and siTET3-treated NCCIT cells (those not accounted for by 5hmC loss in enhancers) are related to functions separate from the TET roles in cytosine methylation patterning described here. The exact nature of these functions is currently unknown, but might be mediated through altered 5hmC levels, changes in 5hmC reader protein localization, or non-enzymatic activities of the TET proteins. Given the lack of knowledge of TET and 5hmC roles in the genome, cancer-specific TET mutations could potentially be directing pathogenic gene expression patterns via any of these routes, something that will be important to examine in future studies. Nonetheless, a link between 5hmC accumulation and gene activation was observed during differentiation of NCCIT cells. Genes with high 5hmC enrichment during differentiation were often abundantly expressed. This was especially true for some ectodermal and mesodermal patterning genes, which were enriched with large peaks of 5hmC during differentiation. Furthermore, genes that were robustly activated during induction of differentiation also tended to be repressed in TET1, TET2, or TET3 depleted cells. Taken together, we propose that TET-mediated enrichment of 5hmC promotes a transcriptionally permissive chromatin environment, and that disruption of this state represents a crucial step toward permanent gene silencing by aberrant DNA methylation in cancer cells.

## Conclusions

The recent elucidation of TET hydroxylation activities on 5mC has changed our view of the epigenome from that of a steady-state methylome to the realization that it is a dynamic and mutable landscape. The results described herein establish a compelling framework for how TET-driven 5hmC patterning impacts gene expression. TET patterning of the epigenome is clearly a common basis of both mammalian development and cellular transformation, and the findings presented here that TETs have multi-dimensional functions in mediating DNA methylation, hydroxymethylation, and gene expression patterns is a crucial step for advancing our mechanistic understanding of how the epigenome functions in both normal and disease states. Overall, this study expands our knowledge of how TET dioxygenases impact cytosine modifications across the cancer genome and reveals that the chromatin landscape and DNA sequence composition significantly influence TET function.

## Materials and methods

### Cell culture, siRNA transfections, and extractions

NCCIT cells (from ATCC) and human H9 (WA09) ESCs were cultured as described [1] and differentiation (NCCIT cells only) was induced by addition of 10 μM all-trans RA (Sigma, St. Louis, MO USA) for 7 days. A172 cells (glioma) were obtained from ATCC and cultured in McCoy’s 5a media containing 10% fetal calf serum. On-TARGETplus SMARTpools (Dharmacon, Thermo Scientific, Lafayette, CO USA) composed of a mixture of four individual siRNAs targeting a single gene were used against *TET1* (L-014635-02), *TET2* (L-013776-03), and *TET3* (L-022722-02) in separate experiments. Transfection with a negative control non-targeting siRNA (D-001206-13-20; Dharmacon, Thermo Scientific) was performed in parallel. For siRNA transfections, approximately 4.5 × 10^4^ NCCIT cells were seeded in each well of a six-well plate. At 24 and 48 hours post-seeding, cells were transfected using PepMute siRNA transfection reagent (SignaGen, Rockville, MD USA) prepared according to the manufacturer’s protocol. Fresh growth medium (900 μl) was added to cells 30 minutes prior to addition of 100 μl of transfection reagent mix. The siRNA transfection mix was composed of 100 μl of PepMute transfection buffer, 1 μl of 40 μM siRNA, and 1.5 μl of PepMute reagent. Fresh media was added to cells at 72 hours post-seeding, and cells were harvested at 96 hours post-seeding. Total RNA was extracted by Trizol homogenization and purified according to the manufacturer’s protocols (Life Technologies, Carlsbad, CA USA). Genomic DNA was extracted by proteinase K digestion and phenol:chloroform extraction as described [66].

### Affinity-based capture of 5hmC and 5mC and sequencing library preparation

Prior to affinity pull-downs, 5 μg of genomic DNA in 130 μl TE was sheared to less than 400 bp on a Covaris S220 focused-ultrasonicator according to the manufacturer’s instructions. Sheared samples were ethanol precipitated and resuspended in TE to a concentration of approximately 350 ng/μl based on nanodrop spectrophometric measurements. Samples were then normalized to the control sample by qPCR standard curves. DNA concentrations were adjusted based on the standard curve. 5hmC enrichment was performed using 2.5 μg of sheared DNA per reaction with the Hydroxymethyl Collector kit according to the manufacturer’s instructions (Active Motif, Carlsbad, CA USA). Each sample was performed in quadruplicate and replicates were pooled after the pull-down prior to preparation of sequencing libraries. Independent 5hmC-capture experiments were performed for 5hmC-qPCR validation experiments. Primers for validation qPCR are from [1] or are listed in Additional file 3. For 5mC-capture, 2 μg of sheared DNA was used as input for the MethylMagnet methylated-CpG DNA isolation kit according to the manufacturer’s instructions (Ribomed, Carlsbad, CA USA) and reactions were performed in quadruplicate for each sample. DNA sequencing libraries were generated from the 5mC and 5hmC captured DNA with the TruSeq DNA sample preparation kit (Illumina, San Diego, CA USA) according to manufacturer’s instructions. Agencourt AMPure XP Beads (Beckman Coulter, Pasadena, CA USA) used during library preparation were calibrated for size selection of DNA fragments greater than 200 bp. PCR amplification of the libraries was performed for 11 cycles. After PCR amplification, the library was gel purified using SYBR gold for visualization of DNA, quantified by qPCR (KAPA Biosystems library quantification kit, Wilmington, MA USA), and analyzed on a bioanalyzer with a high sensitivity DNA chip (Agilent, Santa Clara, CA USA) for quality control and quantification. Libraries were sequenced on an Illumina HiSeq2000 (50 bp read length) at the Tufts University Genomics Core Facility.

### Data analysis

Raw sequencing reads were mapped to the UCSC human genome hg19 build using BWA V0.5.9 [67] with a default parameter setting. Multiply mapped reads and uniquely mapped reads with mismatches and indels >5% of read lengths were filtered out. SICER V1.1 [55] was used to identify enriched regions (peaks) in a sample and differentially enriched regions between two samples relative to an input with the following parameters: redundancy allowed = 1, window size = 200, fragment size = 300, effective genome size = 0.854, gap size = 600, E-value = 1,000, false discovery rate = 0.01. In-house scripts annotated peaks and differentially enriched regions with RefSeq, CGIs, and repeats in the UCSC genome browser [68], and classified them as promoter (-1 kbp to +1 for TSS), body, and 3′ end (TTS + 1 kbp). In some cases, gene bodies were further classified into 5′ UTR, exon, protein coding exon, 3′ UTR, and intron. Genes were also stratified based on the CpG density within their promoter regions (HCPs, intermediate CpG density promoters (ICPs), and LCPs) using the criteria in [69]. In this classification, HCPs are ‘strong’ CGIs while ICPs are ‘weak’ CGIs. LCPs are a distinct class. Gene lists in promoters and bodies were analyzed using in-house scripts via the DAVID server (default settings) for functional annotation using gene ontologies and pathways [70]. After discarding more than two reads mapping to the same location, mapped reads were lengthened to the 3’ end to reflect their original length, and counted based on their midpoint for genomic features such as genes, CGIs, and repeats. A genomic feature was binned by relative positions including upstream and downstream regions. Different numbers of mapped reads per sample were taken into account by calculating FPKM (fragments per kilobase per million fragments mapped). To illustrate the change in tag densities around genes, we used a relative length window for gene bodies and measured the average of normalized read coverage in a window.

### 5mC and 5hmC quantification and TAB-seq

DNA methylation and hydroxymethylation quantification was performed using the MethylFlash methylated and hydroxymethylated colorimetric DNA quantification kits (P-1034; p-1036; Epigentek, Farmingdale, NY USA) according to the manufacturer’s instructions. All samples were run in triplicate. TAB conversion of DNA was performed as described [71]. To accommodate for Sanger sequencing, DNA was sheared with a Covaris S220 to less than 10 kb in size and purified by ethanol precipitation prior to TAB conversion. Bisulfite conversion and sequencing of DNA were performed as previously described [1]. Up to 12 independent clones were sequenced for each region. Primer sequences are listed in Additional file 3. TAB-seq plots were generated with QUMA [72].

### Expression analysis by qRT-PCR and microarray

CDNA synthesis, qRT-PCR, and data analysis was performed as described previously [73,74]. qRT-PCR primers were designed and selected for optimal efficiency based on their performance with a standard curve of cDNA template. qRT-PCR was performed with at least three replicates. Primer sequences are listed in Additional file 3. Gene expression profiling was performed using Affymetrix Human Gene 1.0 ST arrays. All samples were analyzed in duplicate at the Georgia Regents University Cancer Center Genomics Core facility as described previously [1].

### Gene ontology analysis and statistical methods for data set comparisons

Ontology analysis was performed using the functional annotation tool within the DAVID bioinformatics database [70,75]. Fisher exact test with a two-tailed *P*-value calculation was used for testing the significance of data set comparisons as described previously for similar data sets [76]. For added stringency, a modified EASE score was applied to all Fisher exact tests [70,75].

### Data access

Sequencing and expression microarray data have been deposited into the NCBI Gene Expression Omnibus database under accession number GSE51903. Additional published datasets used in this analysis include: GSM747152, GSM605307, and GSE38938.

## Abbreviations

5hmC: 5-hydroxymethylcytosine; 5mC: 5-methylcytosine; bp: base pair; CGI: CpG island; DF: differentiated by retinoic acid; DNMT: DNA methyltransferase; ECC: embryonic carcinoma cell; ESC: embryonic stem cell; H2AK119ub: histone H2A lysine 119 ubiquitination; H3K27ac: histone H3 lysine 27 acetylation; H3K27me3: histone H3 lysine 27 trimethylation; H3K36me3: histone H3 lysine 36 trimethylation; H3K4me3: histone H3 lysine 4 trimethylation; HCP: high CpG density promoter; ICP: intermediate CpG density promoter; LCP: low CpG density promoter; NTC: non-targeting control; PCR: polymerase chain reaction; qPCR: quantitative PCR; RA: retinoic acid; siRNA: small interfering RNA; TAB: TET-assisted bisulfite conversion; TET: Ten-eleven translocation; TSS: transcription start site; TTS: transcription termination site; UD: undifferentiated pluripotent state; UTR: untranslated region.

## Competing interests

The authors declare that they have no competing interests.

## Authors’ contributions

ELP coordinated design of the study, conducted the majority of experiments and data/statistical analysis, and drafted the manuscript. RLT, JJT, and CL generated and analyzed data. TH edited the manuscript and provided intellectual input. J-HC performed the sequence alignment, peak calling, differential peak analysis, and coordinated the data and statistical analyses. KDR conceived of the study and its design, coordinated the data and statistical analyses, and edited the manuscript. All authors read and approved the final manuscript.

## Supplementary Material

Additional file 1 Supplementary figures. Click here for file

Additional file 2: Table S1 Number of differentially methylated genes stratified by magnitude of methylation change. Click here for file

Additional file 3: Table S2 PCR primer sequences. Click here for file
