# Supplementary material for: Distinct and overlapping control of 5-methylcytosine and 5-hydroxymethylcytosine by the TET proteins in human cancer cells
Source: Genome Biol. 2014 Jun 23;15(6):R81. doi: 10.1186/gb-2014-15-6-r81 (PMC4197818; doi:10.1186/gb-2014-15-6-r81)
Supplement: Additional file 1 — Supplementary figures. [file gb-2014-15-6-r81-S1.pdf]

Additional File 1 – Supplementary Figures

Putiri *et al*., Distinct and overlapping control of 5-methylcytosine and 5-hydroxymethylcytosine by the TET proteins in human cancer cells

Supplementary Fig 1

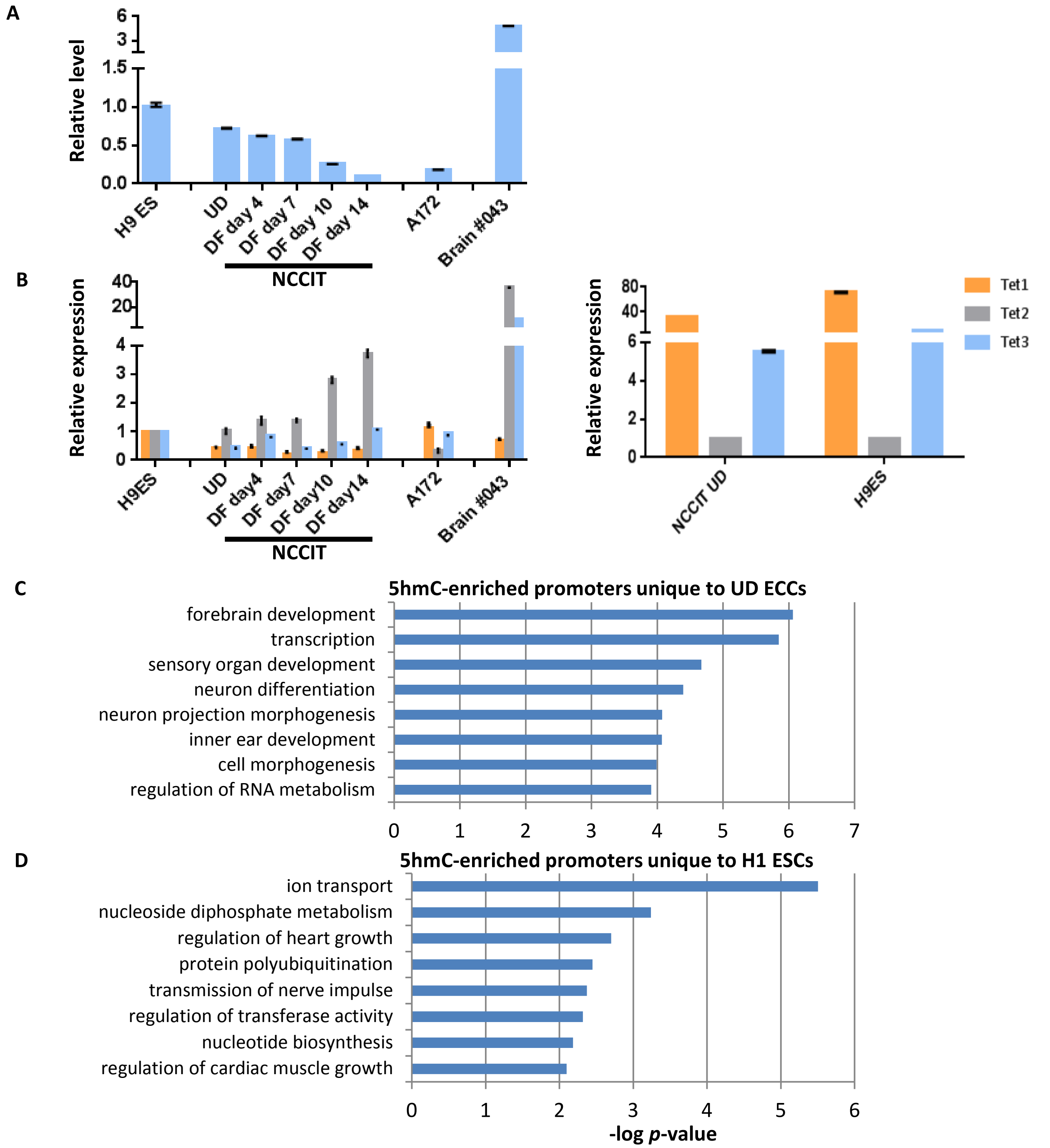

Supplementary Figure 1. Comparison of 5hmC levels and localization, and TET expression patterns, in NCCIT embryonic carcinoma cells relative to human embryonic stem cells. (A) Quantification of total genomic 5hmC in the indicated cell lines or tissues. All samples were run in triplicate and values are set relative to H9 hES (at 1.0). (B) Left. Quantification of TET1, TET2, and TET3 RNA level by qRT-PCR in the indicated cell lines and tissues. Samples were run in triplicate and values are set relative to H9 hESC (at 1.0) and normalized to GAPDH and dynein. Right. Expression of TET1, TET2, and TET3 in UD NCCIT and H9 ESC, set relative to TET2 (at 1.0) and normalized to GAPDH and dynein to illustrate the expression of each TET relative to the other TETs in each cell line. DAVID ontology analysis was performed on (C) promoters with 5hmC enrichment only in NCCIT UD but not H1 ESC and (D), promoters with 5hmC enrichment only in H1 ESCs.

Supplementary Fig 2

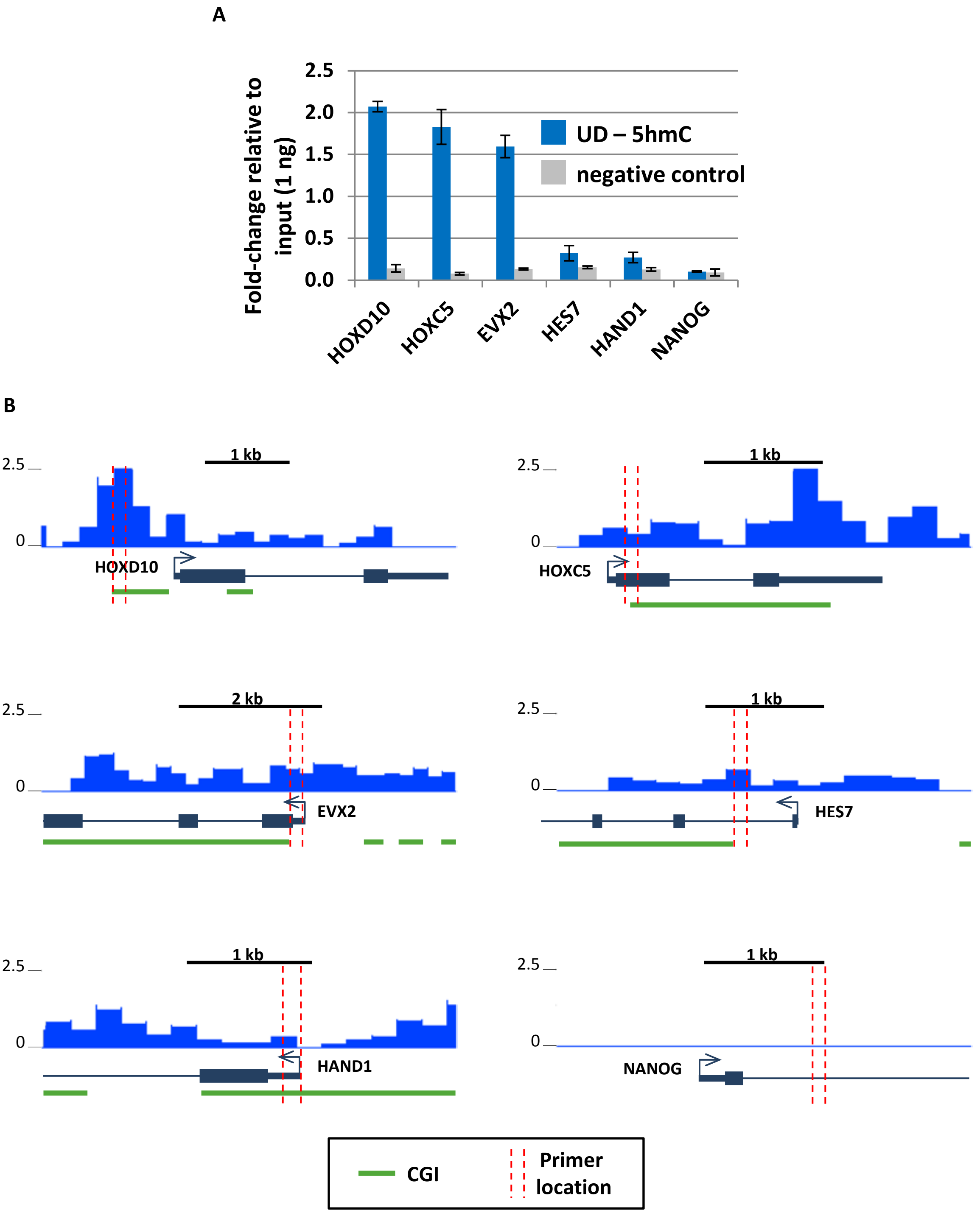

Supplementary Figure 2. Independent confirmation of 5hmC-seq results by 5hmC pull-down coupled with QPCR. (A) Enrichment of 5hmC by qPCR in NCCIT UD relative to input for the indicated regions. (B) 5hmC-seq results for each PCR-amplified region based on the UDP-azide-glucose labeling, capture, and deep sequencing method. Bent arrow = promoter.

Supplementary Fig 3

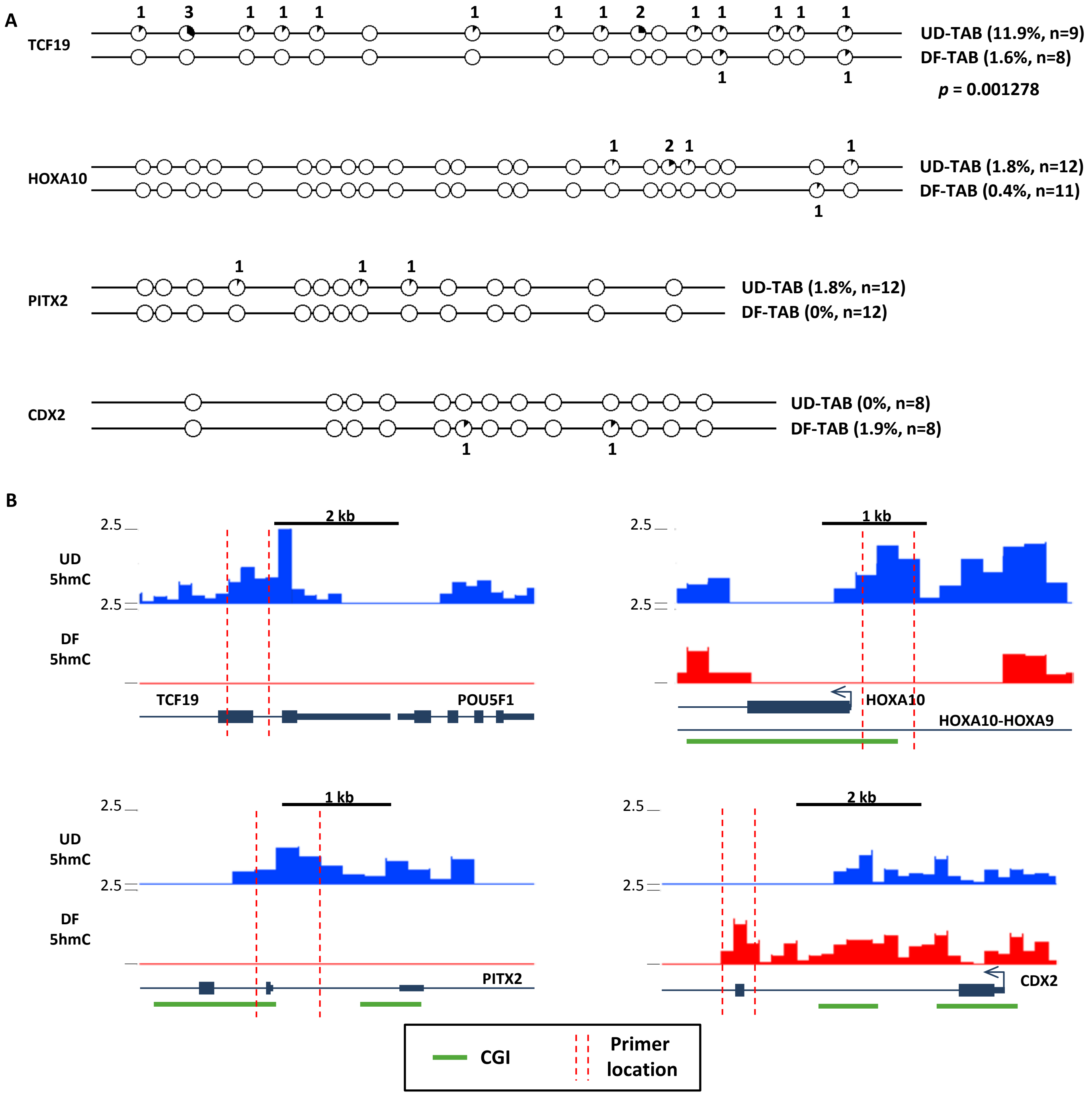

Supplementary Figure 3. Independent confirmation of 5hmC-seq results by TET-assisted bisulfite (TAB) conversion coupled with Sanger sequencing. (A) Base pair resolution of 5hmC by TAB-sequencing. Each pie chart represents a CpG dinucleotide with the black portion denoting the proportion of all cytosines that are 5hmC (the number of 5hmC nucleotides at the given CpG site is noted above or below each pie). The total percentage of 5hmC nucleotides within the given region and the total number (n) of clones sequenced for each sample are denoted on the right. (B) 5hmC-seq results for each TAB-sequenced region. Bent arrow=promoter.

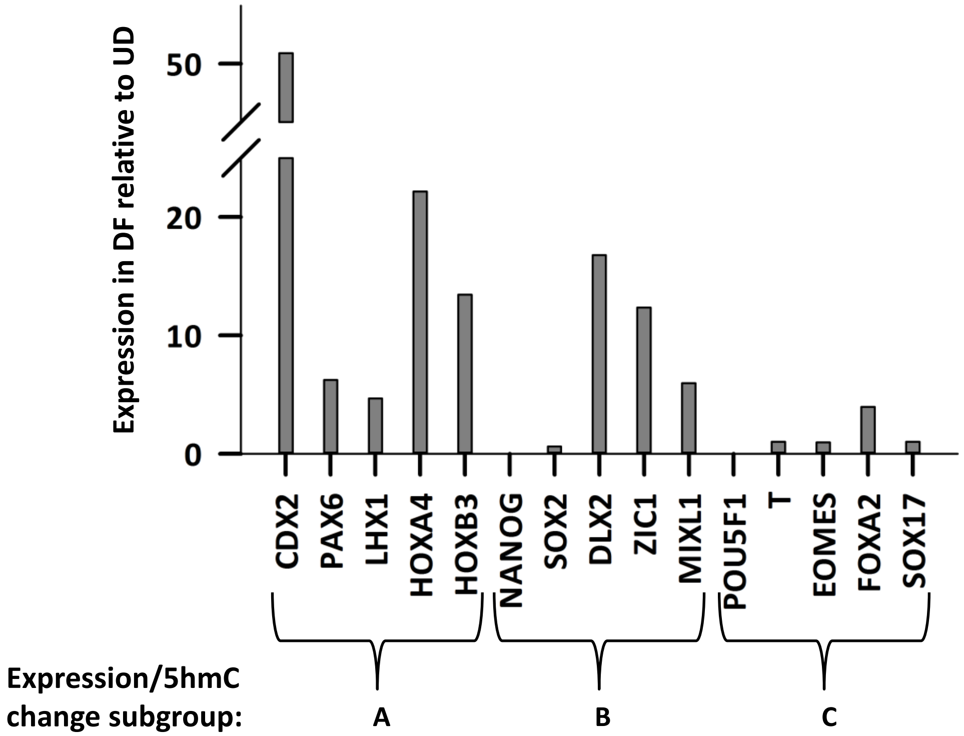

Supplementary Figure 4. Three subgroups characterize intragenic 5hmC changes in NCCIT cells after differentiation. (A) This subgroup of genes exhibited large 5hmC accumulation and increased expression upon induction of differentiation. Representatives of this subgroup include genes involved in ectodermal and some mesodermal differentiation and in embryonic patterning, including portions of the *HOXA* and *HOXB* clusters. (B) The second subgroup of genes showed variable loss and redistribution of 5hmC. Many mesodermal and ectodermal lineage-specific genes that become activated during differentiation and the pluripotency markers *NANOG* and *SOX2* whose expression decreases in DF were represented in this second subgroup. (C) The third subgroup of 5hmC changes in DF were defined by total 5hmC loss or near complete 5hmC loss. These genes include some of the earliest determinants of endodermal and mesodermal fates. The pluripotency factor *POU5F1*/*Oct4* (which is also suggested to function in endoderm specification [1, 2]) is among these markers. These genes are expressed at low levels in DF. Expression is shown as DF relative to NCCIT UD cells and is based on expression microarray data (A-C).

Supplementary Fig 5

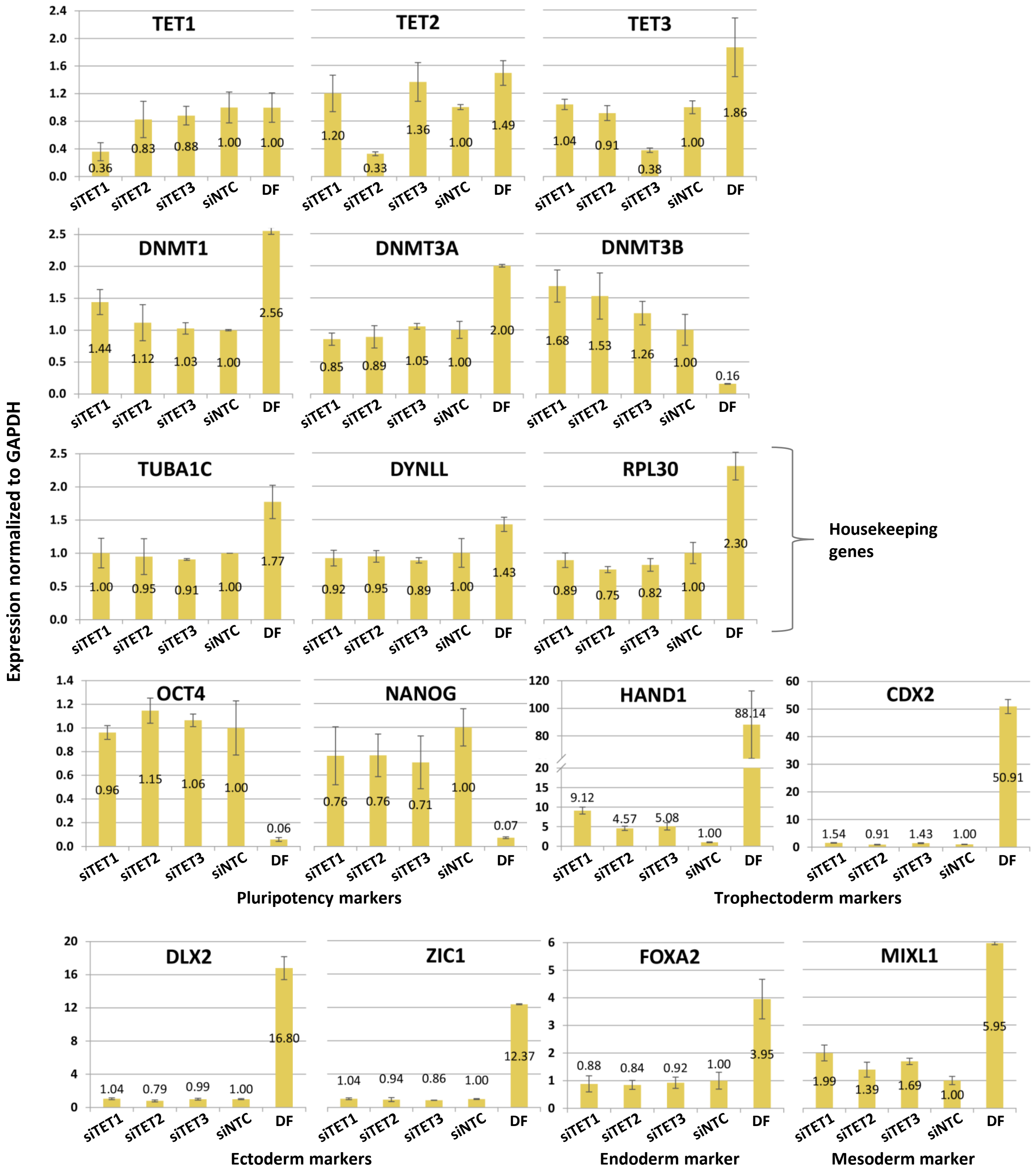

Supplementary Figure 5. Validation of the experimental system for TET1, TET2, and TET3 depletion in NCCIT UD cells. Expression of TETs, DNMTs, housekeeping genes, pluripotency markers, and differentiation markers normalized to GAPDH and relative to siNTC in each TET depletion or upon differentiation induction (DF) by retinoic acid in NCCIT cells.

Supplementary Fig 6

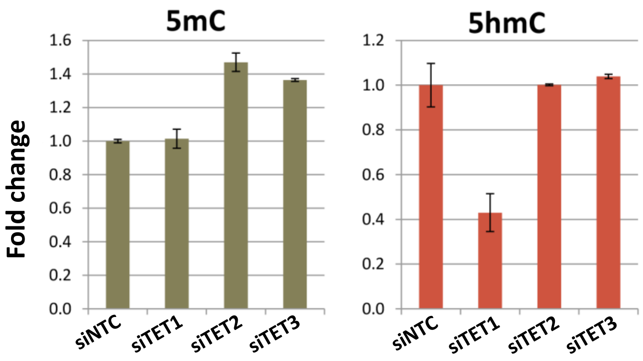

Supplementary Figure 6. Effects of siTET1, siTET2, and siTET3 on total 5mC and 5hmC levels. Global quantification of 5mC and 5hmC for siTET treated cells showed a moderate increase of 5mC in siTET2 and siTET3 (although this did not reach statistical significance). siTET1 cells showed a significant (p = 0.0258) ~60% depletion of genomic 5hmC relative to siNTC (set at 1.0).

Supplementary Fig 7

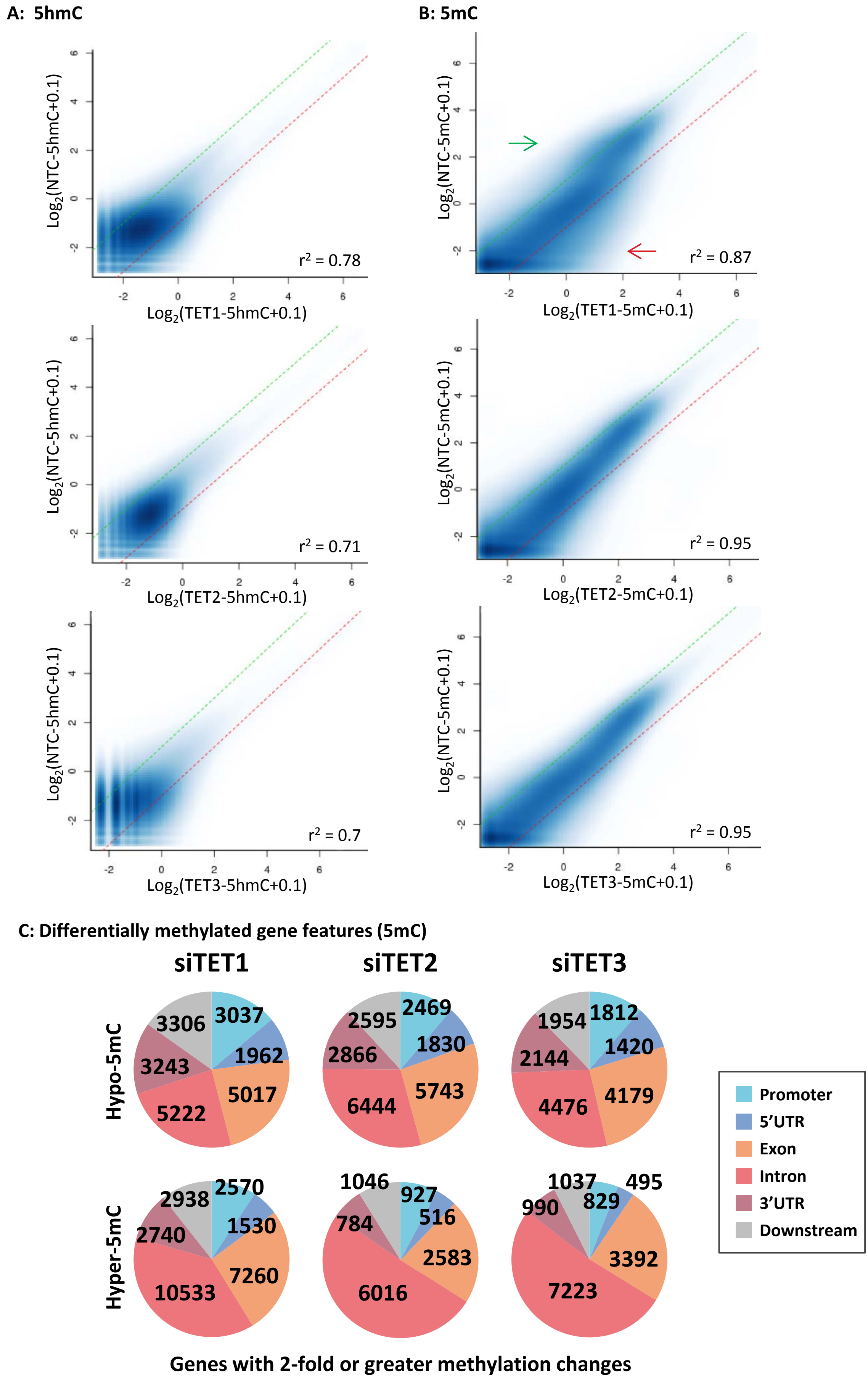

Supplementary Figure 7. 5mC and 5hmC in siTET depletions compared to siNTC. Scatterplots for peaks of 5hmC (A) and 5mC (B) called by SICER [3]. Methylation enrichment scores for siTET samples and siNTC are plotted on the X-axis and Y-axis, respectively. Dotted green and red lines represent limits for 2-fold hypo- and hypermethylation, respectively. Pearson's correlation coefficient is shown. (C) Pie charts for genes with decreased (top) and increased (bottom) 5mC. Pie pieces represent total number of genes with 2-fold or greater 5mC change in the specified gene region.

Supplementary Fig 8

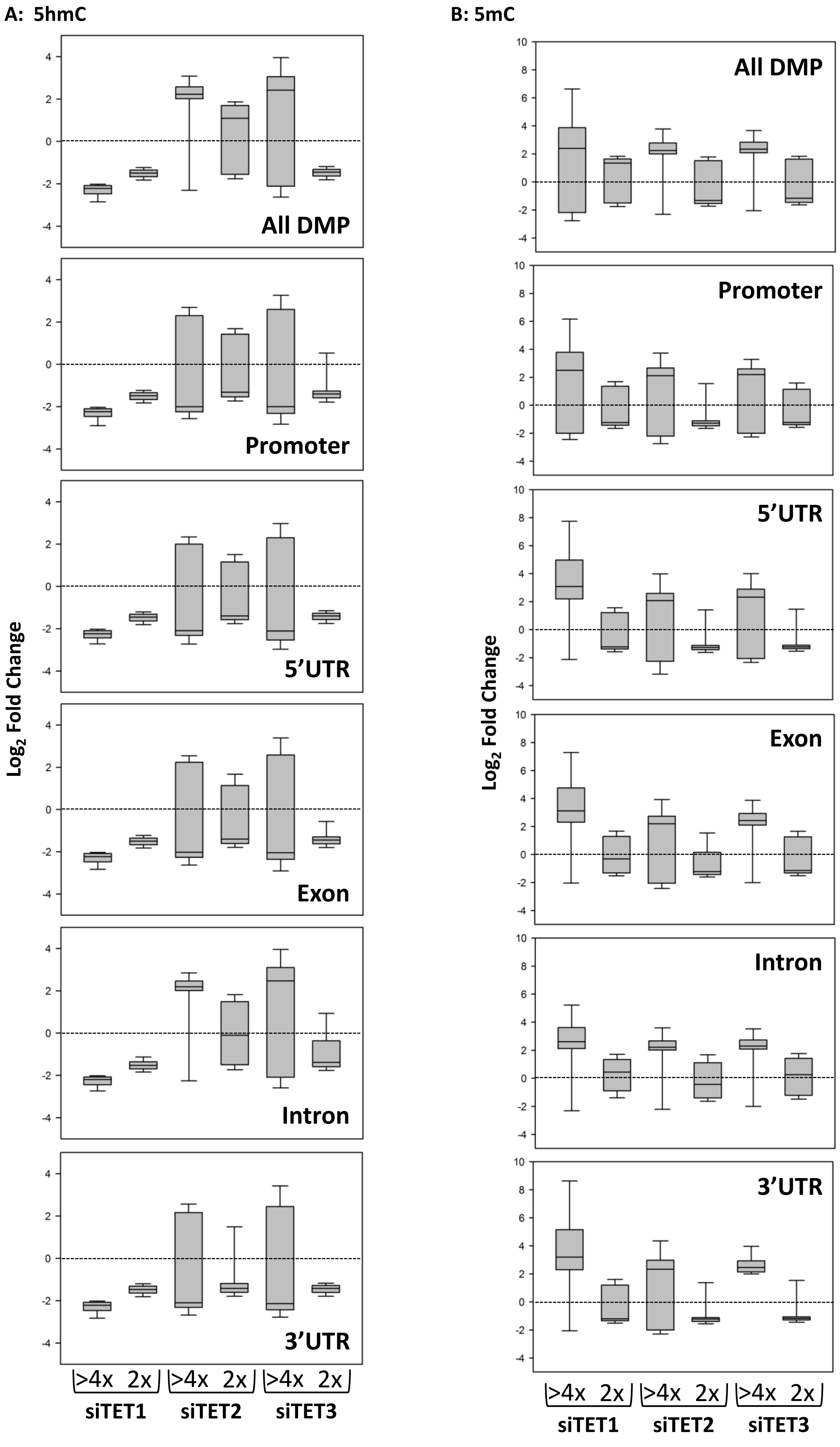

Supplementary Figure 8. Stratification of epigenetic changes by magnitude. Box plots for log_2 fold-change based on differential SICER analysis of 5hmC (left, A) and 5mC (right, B) peaks. Fold-change is shown for all differentially methylated peaks (DMP) and specific gene regions as marked. Fold-change is stratified by changes of greater than 4-fold (>4x) and changes between 2-fold and 4-fold (2x).

Supplementary Fig 9

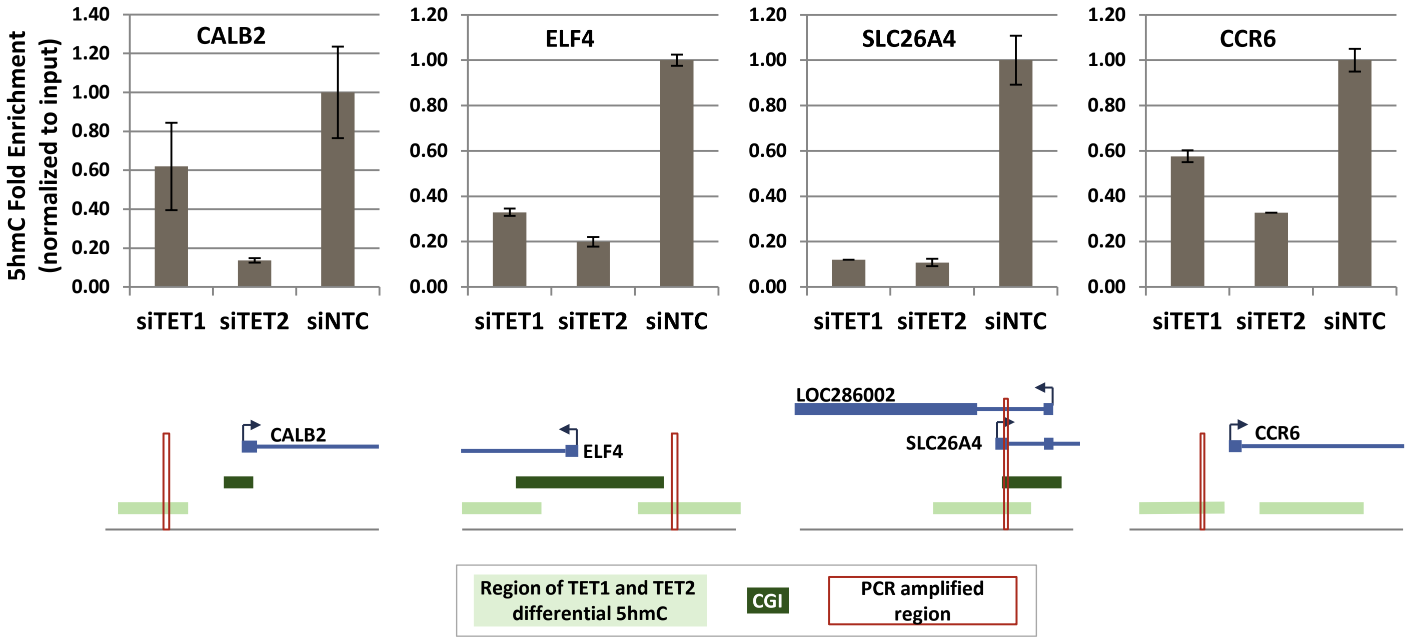

Supplementary Figure 9. Independent confirmation of siTET-mediated DNA hydroxymethylation changes by 5hmC pull-down coupled with QPCR. Graphs show enrichment of 5hmC normalized to input and relative to siNTC. Beneath each graph is a schematic representation of the genetic region with 5hmC depletion (based on deep sequence analysis) in siTET1 and siTET2. Light green bars indicate the region with reduced 5hmC called by SICER, dark green bars show location of CpG islands, and red lines mark the location of PCR primers and region amplified by PCR.

Supplementary Fig 10

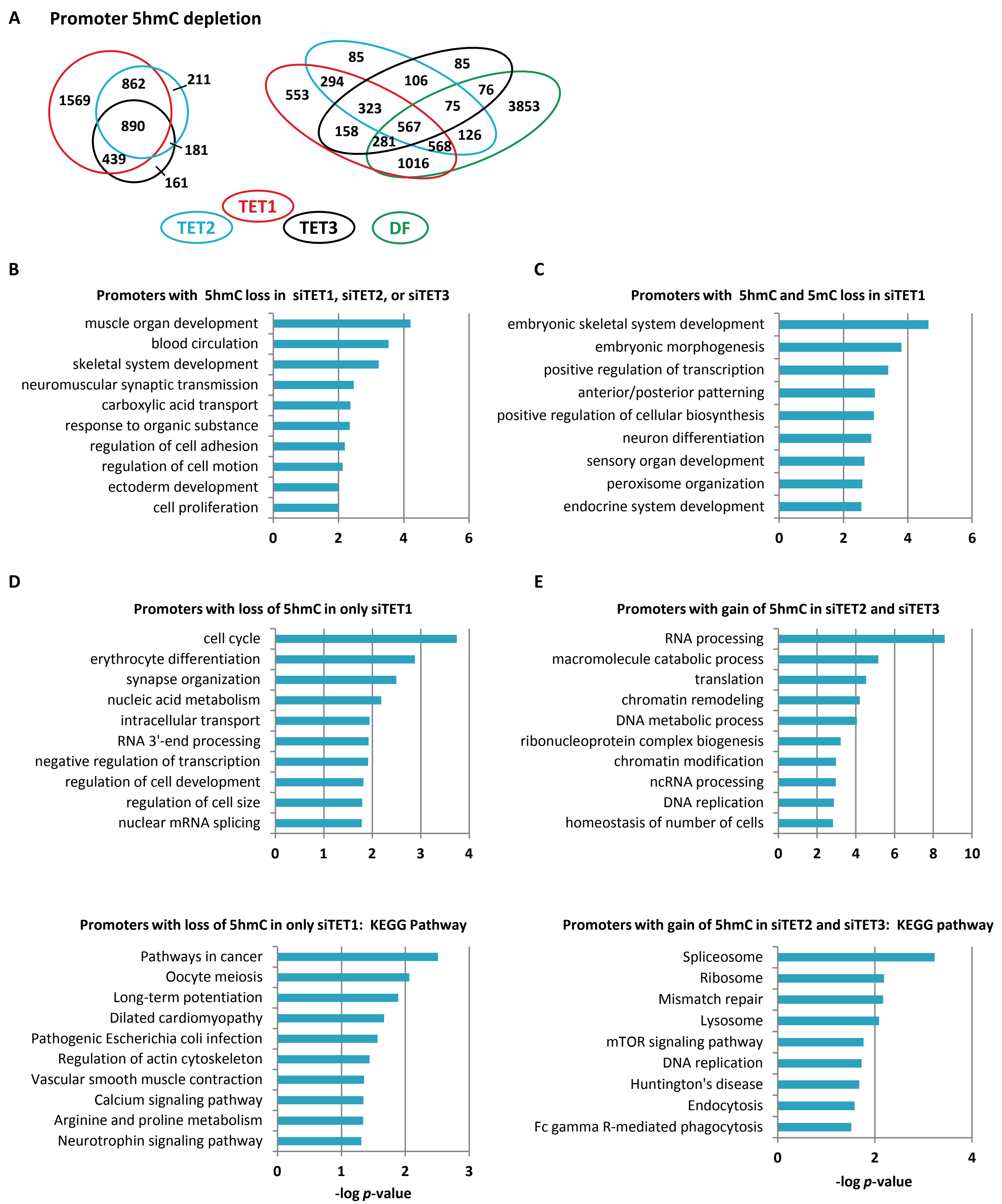

Supplementary Figure 10. Impact of TET depletions on 5hmC levels in promoters. (A) Overlap of promoters with loss of 5hmC among siTETs (left) ($p < 0.0001$) and among siTETs and differentiated NCCIT cells (DF, right) ($p < 0.0001$). Ontology analysis of: (B) genes with promoter 5hmC reduction in any of the three siTET1, siTET2, and siTET3 single depletions, (C) genes with promoter 5hmC and 5mC reduction in siTET1, (D) genes that are unique targets of siTET1, and (E) genes with hyper-5hmC in siTET2 and siTET3. Bottom charts in (D) and (E) represent KEGG pathways affected.

Supplementary Fig 11

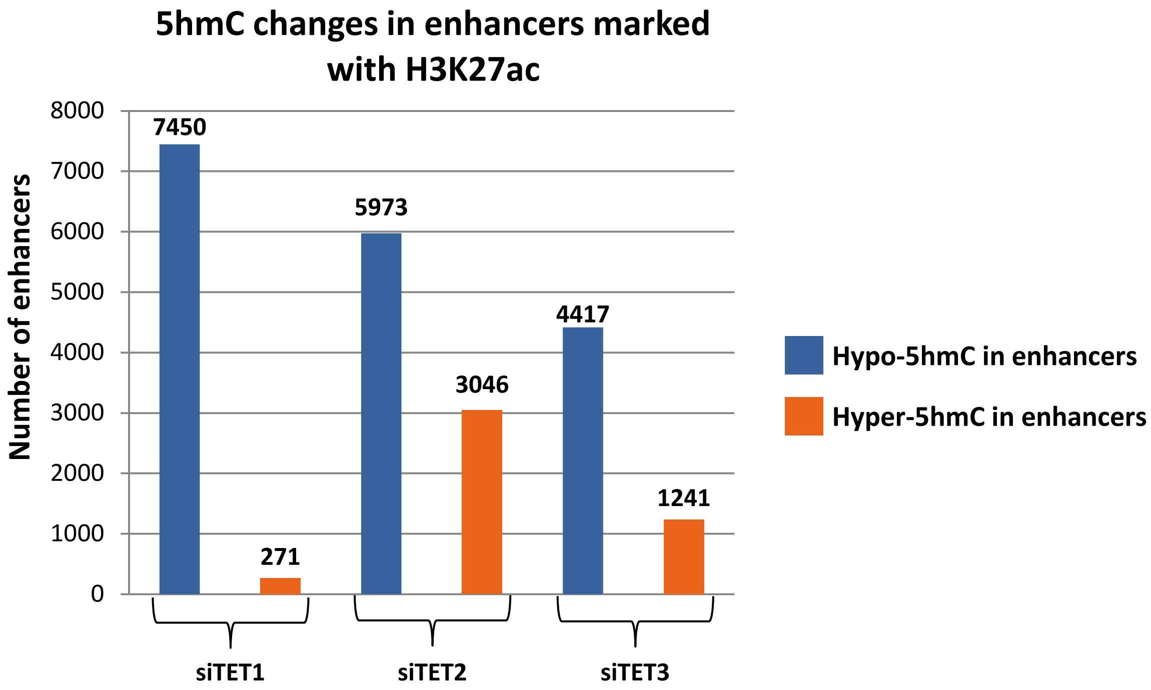

Supplementary Figure 11. H3K27ac-marked enhancers become depleted of 5hmC in TET-depleted cells. Number of enhancers with loss or gain of 5hmC in siTET depleted cells.

Supplementary Fig 12

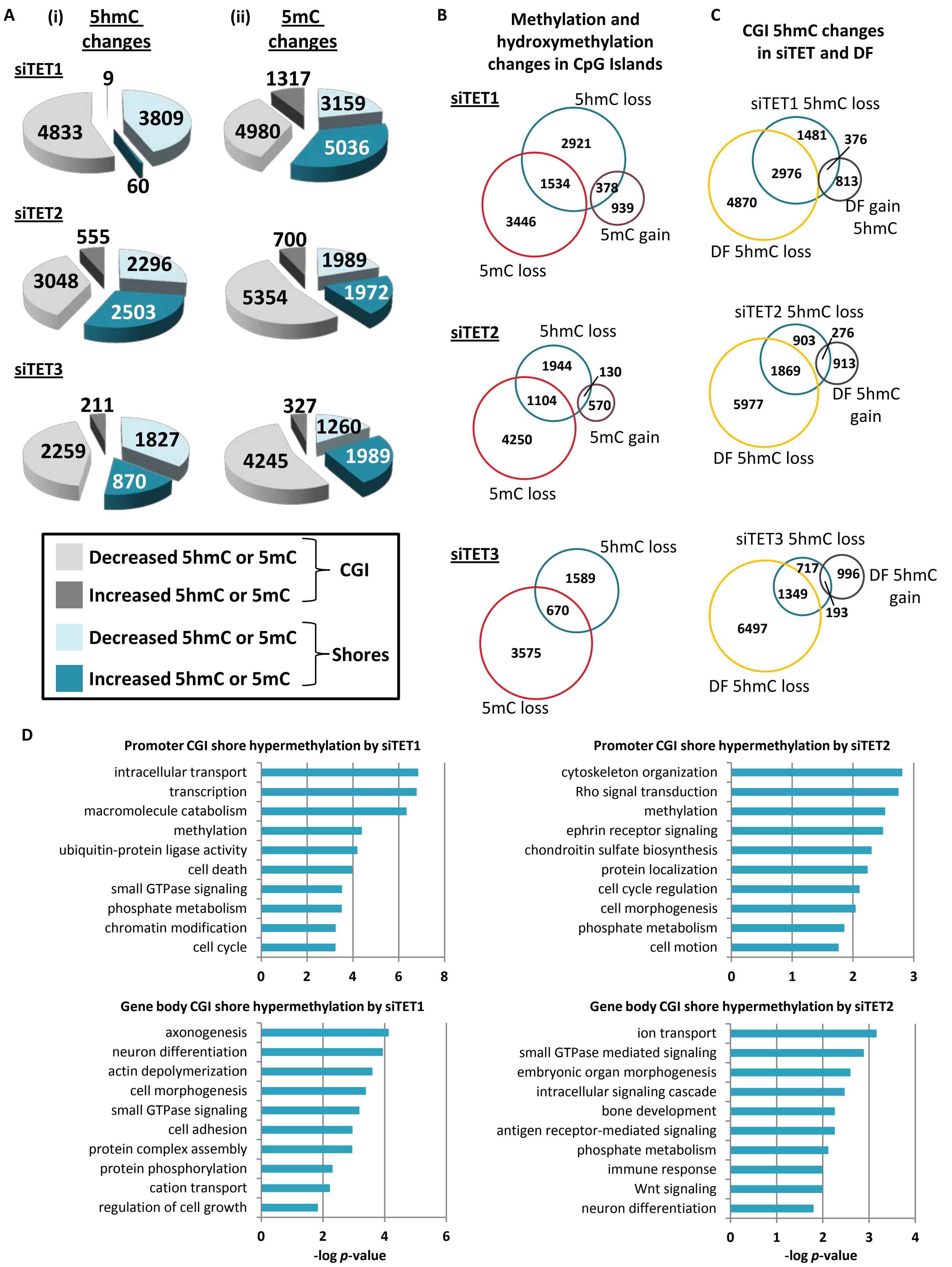

Supplementary Figure 12. Impact of TET depletion on cytosine modifications in CGI and CGI shores. (A) Pie charts representing the number of CGI or shores with increased or decreased 5hmC and 5mC. (B) CGI with 5hmC loss, 5mC loss, and 5mC gain overlap significantly ($p < 0.0001$). (C) Loss of 5hmC in siTET1, siTET2, and siTET3 overlap significantly with 5hmC changes that occur upon differentiation (DF, $p < 0.0001$). (D) Ontology analysis for promoters and gene bodies with CGI shore hypermethylation in siTET1 and siTET2.

Supplementary Fig 13

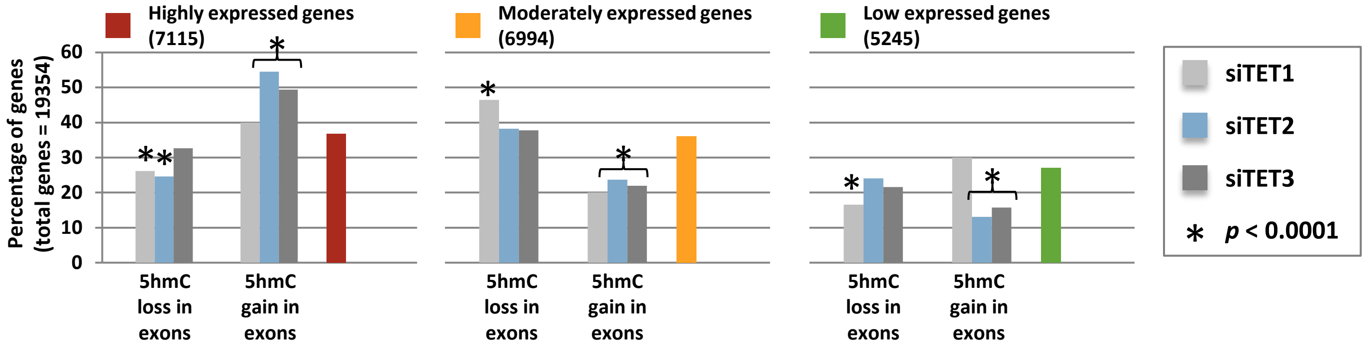

Supplementary Figure 13. Connection between basal level of gene expression and TET epigenetic function in exons. Hypo- or hyper-5hmC exons of siTET depletions were compared to basal gene expression levels in UD NCCIT. Shown is the percentage of genes with 5hmC changes in exons in siTET that occur in highly, moderately, or low expressing genes. These percentages are compared to the total percentage of highly (red bar), moderately (gold bar), or low (green bar) expressing genes.

Supplementary Fig 14

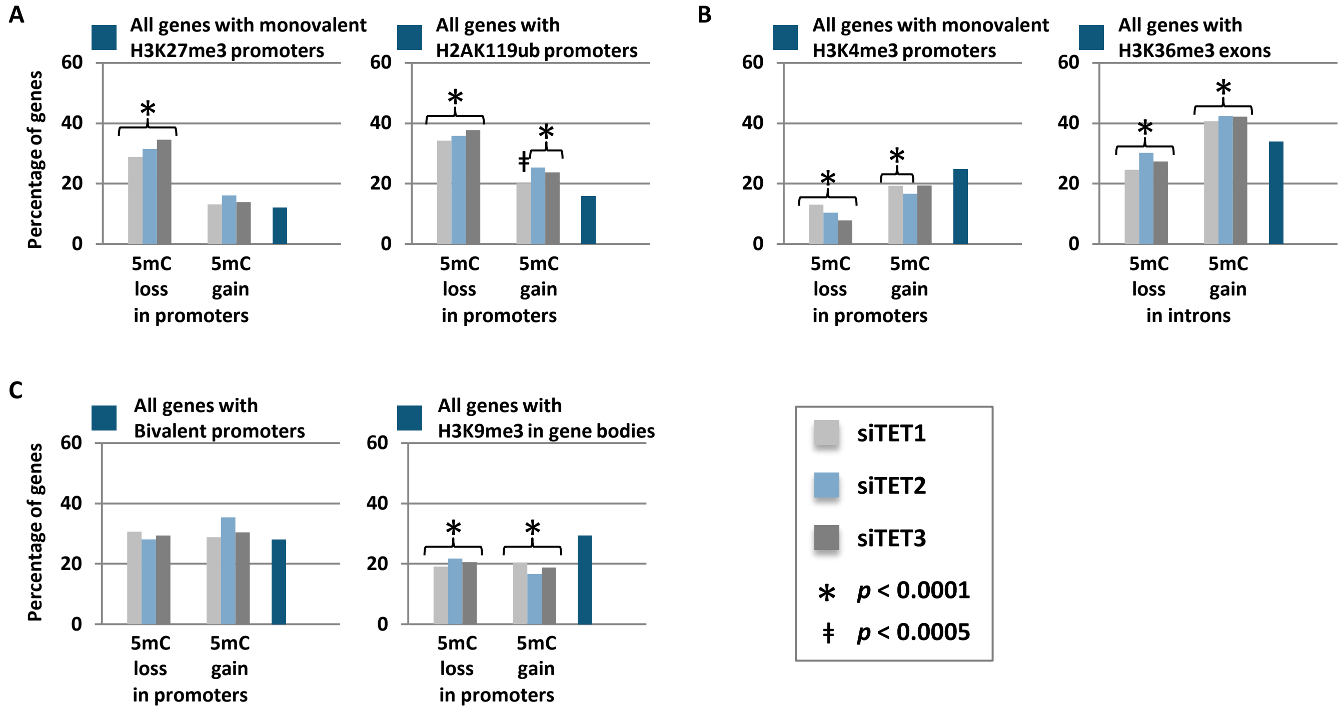

Supplementary Figure 14. Relationships between cytosine modification changes and histone mark occupancy. (A-C) Genes with hypomethylation or hypermethylation were compared to subsets of genes with histone modifications (as marked) mapped previously in UD NCCIT [4]. Shown are the percentage of genes with 5mC changes that are marked by the given histone modification, and the percentage of all promoters with the given histone modification in the genome (total number of genes = 23218; total genes with 5mC changes per region are as listed in Supp. Fig. 7C). Promoters with 5mC changes that have an overrepresentation or underrepresentation of the given histone mark are designated with * when $p < 0.0001$ or ǂ when $p < 0.0005$.

SUPPLEMENTARY REFERENCES

1. Lunde K, Belting HG, Driever W: **Zebrafish pou5f1/pou2, homolog of mammalian Oct4, functions in the endoderm specification cascade.** *Curr Biol* 2004, **14:**48-55.
2. Aksoy I, Jauch R, Chen J, Dyla M, Divakar U, Bogu GK, Teo R, Leng Ng CK, Herath W, Lili S, et al: **Oct4 switches partnering from Sox2 to Sox17 to reinterpret the enhancer code and specify endoderm.** *Embo J* 2013, **32:**938-953.
3. Zang C, Schones DE, Zeng C, Cui K, Zhao K, Peng W: **A clustering approach for identification of enriched domains from histone modification ChIP-Seq data.** *Bioinformatics* 2009, **25:**1952-1958.
4. Jin B, Ernst J, Tiedemann RL, Xu H, Sureshchandra S, Kellis M, Dalton S, Liu C, Choi JH, Robertson KD: **Linking DNA methyltransferases to epigenetic marks and nucleosome structure genome-wide in human tumor cells.** *Cell Rep* 2012, **2:**1411-1424.
