# Supplementary material for: Distinct and overlapping control of 5-methylcytosine and 5-hydroxymethylcytosine by the TET proteins in human cancer cells
Source: Genome Biol. 2014 Jun 23;15(6):R81. doi: 10.1186/gb-2014-15-6-r81 (PMC4197818; doi:10.1186/gb-2014-15-6-r81)
Supplement: Additional file 2: Table S1 — Number of differentially methylated genes stratified by magnitude of methylation change. [file gb-2014-15-6-r81-S2.pdf]

**Supplementary Table 1. Number of differentially methylated genes stratified by magnitude of methylation change**

| | Methylation Change | 5'End | | 5'UTR | | Exon | | Intron | | 3'UTR | |
|---|---|---|---|---|---|---|---|---|---|---|---|
| | | Hypo- | Hyper- | Hypo- | Hyper- | Hypo- | Hyper- | Hypo- | Hyper- | Hypo- | Hyper- |
| TET1 | 2-4 fold | 3037 | 2570 | 1962 | 1530 | 5017 | 7260 | 5222 | 10533 | 3243 | 2740 |
| | > 4 fold | 338 | 988 | 100 | 620 | 434 | 3514 | 692 | 3484 | 168 | 1325 |
| TET2 | 2-4 fold | 2469 | 927 | 1830 | 516 | 5743 | 2583 | 6444 | 6016 | 2866 | 784 |
| | > 4 fold | 169 | 262 | 85 | 116 | 234 | 575 | 335 | 1250 | 63 | 187 |
| TET3 | 2-4 fold | 1812 | 829 | 1420 | 495 | 4179 | 3392 | 4476 | 7223 | 2144 | 990 |
| | > 4 fold | 75 | 217 | 47 | 124 | 114 | 974 | 177 | 1585 | 34 | 318 |
