# Supplementary material for: Distinct and overlapping control of 5-methylcytosine and 5-hydroxymethylcytosine by the TET proteins in human cancer cells
Source: Genome Biol. 2014 Jun 23;15(6):R81. doi: 10.1186/gb-2014-15-6-r81 (PMC4197818; doi:10.1186/gb-2014-15-6-r81)
Supplement: Additional file 3: Table S2 — PCR primer sequences. [file gb-2014-15-6-r81-S3.pdf]

Supplementary Table 2. PCR primer sequences

QRTPCR primers

TET1	GGGCACCCTACCGACAGAAGATGC	CTTCTGGGGCTTGGGCTTCTACC
TET2	GGATGTCCTATTGCTAAGTGG	GAATCACAATCACTGCAGCCTC
TET3	GAGCTGGCGGGCATTACG	TGCGGCTCCACCTTGAGG
GAPDH	CTTTGGTATCGTGGAAGGACTC	GTAGAGGCAGGGATGATGTTC
DNMT1	GGAGAGGCTAAGCGTTCAAG	AAATGAGATGTGATGGTGGTTTG
DNMT3A	AAGAGCACAGCGGAGAAG	GCAGATGTCCTCAATGTTCC
DNMT3B	CCATGAAGGTTGGCGACAA	TGGCATCAATCATCACTGGATT
TUBA1C	CCGGGCAGTGTTTGTAGACTTGG	ATCTCCTTGCCAATGGTGTAGTGCC
DYNLL	CATAGAGAAGGACATTGCGGCTCATATC	GAACAGAAGAATGGCCACTTGGC
RPL30	TTGAACTGGGCACAGCATGCGG	CTTTTCACCAGTCTGTTCTGGCATGC
OCT4/POU5F1	CTTGCTGCAGAAGTGGGTGGAGGAA	CTGCAGTGTGGGTTTCGGGCA
NANOG	GAACTCTCCAACATCCTGAACC	TTCTGCGTCACACCATTGC
HAND1	AAAGGCTCAGGACCCAAGAA	CAGCACGTCCATCAGGTAGG
CDX2	GCTACATCACCATCCGGAG	GCTGCTGCAACTTCTTCTTG
DLX2	AGACTCAATACTTGGCCTTGC	GCGAAGCACAAGGTGGAGAAGCG
ZIC1	CAAGTCCTACACGCATCCCAG	CGTGGAGGATTCGTAGCCAGAG
FOXA2	TATGCTGGGAGCGGTGAAGATGG	CGTGTTCATGCCGTTCATCC
MIXL1	CAAGCGCACGTCTTTCAGC	GCACAGTGGTTGAGGATAATCT

Genomic QPCR primers for 5hmC-PCR

CALB2	CGTTGTTCTCTGTCCACTG	CAGAACTAGATCAACCCGTACCA
ELF4	CTGGGGTGACTGGAGAGTTTCGG	CCTTCCCCTGACTCATATCTTGCC
SLC26A4	CTCTTAAGTGGTCACGGATCAG	GTCTCTCCCCTCGTCCTGTTTTCC
CCR6	GGCTCATACATTCTTGGGAAGTTAC	GGACATTGTGACCTTCATTTTGCAG

Bisulfite primers for TAB

TCF19 / POU5F1	TGGTTTTGAAGGGTTTTTAGGAATTAG	AACTCCACTTACCCAATAAAAATC
HOXA10	GATATAAATATGTAATTTGTATTGATG	CTAATTCACTCTAAACTCCTAACACT
PITX2	GTAGGAGAAGGGGGTTTTTATTTAATTG	AACTTTATAATCCTCTAACCTCTAAACAC
CDX2	TAGTTTGGGTTATAAAGTAAGAT	CTTACAATTCTCAACCCTCACTTCTC
